# Evidence and quantification of memory effects in competitive first-passage events

**DOI:** 10.1126/sciadv.adp2386

**Published:** 2025-03-21

**Authors:** Maxim Dolgushev, Toni Vieira Mendes, Benjamin Gorin, Kaili Xie, Nicolas Levernier, Olivier Bénichou, Hamid Kellay, Raphaël Voituriez, Thomas Guérin

**Affiliations:** ^1^ Laboratoire de Physique Théorique de la Matière Condensée, CNRS/Sorbonne University, 4 Place Jussieu, 75005 Paris, France.; ^2^ Laboratoire Ondes et Matière d’Aquitaine, CNRS/University of Bordeaux, F-33400 Talence, France.; ^3^ CINaM, CNRS/Aix Marseille Univ, Marseille, France.; ^4^ Laboratoire Jean Perrin, CNRS/Sorbonne University, 4 Place Jussieu, 75005 Paris, France.

## Abstract

Splitting probabilities quantify the likelihood of a given outcome out of competitive events. This key observable of random walk theory, historically introduced as the gambler’s ruin problem, is well understood for memoryless (Markovian) processes. However, in complex systems such as polymer fluids, the motion of a particle should typically be described as a process with memory, for which splitting probabilities are much less characterized analytically. Here, we introduce an analytical approach that provides the splitting probabilities for one-dimensional isotropic non-Markovian Gaussian processes with stationary increments, in the case of two targets. This analysis shows that splitting probabilities are controlled by the out-of-equilibrium trajectories observed after the first passage. This is directly evidenced in a prototypical experimental reaction scheme in viscoelastic fluids. These results are extended to *d*-dimensional processes in large confining volumes, opening a path toward the study of competitive events in complex media.

## Teaser

Theory and experiments in viscoelastic fluids reveal and quantify the impact of memory on the outcome of competitive events.

## INTRODUCTION

**Fig. 1.**
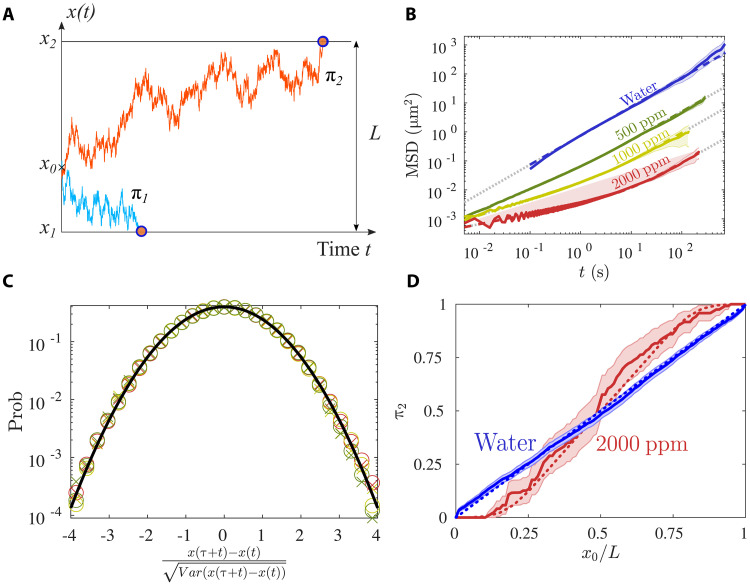
Competitive events and non-Markovian motion. (**A**) Sketch of the problem of competitive events investigated in this paper: In the presence of two targets, for a random walk, what is the probability to hit one target before the other? (**B**) Experimental MSD of tracer particles of 1 μm diameter in viscoelastic fluids, with polymer concentrations (from top to bottom) *c* = 0 ppm (blue), *c* = 500 ppm (green), *c* = 1000 ppm (yellow), and *c* = 2000 ppm (red). The dashed lines are fits using Eq. 9. (**C**) Normalized histograms of the increments x(t+τ)−x(t) for $\tau =\tau_{0}$ (crosses) and $\tau =\tau_{0}/2$ (circles) for the three polymer concentrations of B (with the same color code). Here, $\tau_{0}$ is the memory time of the solution defined in Eq. 9, with $\tau_{0}≃1.48$ s for c=500 ppm, $\tau_{0}≃2.9$ s for c=1000 ppm, and $\tau_{0}≃7$ s for c=2000 ppm. The black line is the density of a normalized Gaussian, $p\left(x\right)=e^{-x^{2}/2}/\sqrt{2\pi }$. (**D**) Values of the splitting probabilities measured for beads in water (blue curves) and in viscoelastic fluids at c=2000 ppm (red) in our experiments and theory (dotted lines), for *L* = 0.6 μm. Error bars are twice the SD of the mean, calculated with n=116 recorded first-passage events.

Which will you reach first: fortune or ruin? It is ubiquitous that the fate of a system depends on which of a finite set of possible outcomes is realized first (see Fig. 1A). A historical example is provided by the “gambler’s ruin problem,” in which one wishes to know the risk for a gambler to go bankrupt before making a given profit (*1*). A form of this problem can be traced back to Pascal, and quantifying the risk of ruin has become a classical problem in financial mathematics (*2*). Competitive events appear in various domains and under different names; examples include the fixation probability of a mutant in the context of population dynamics (*3*) or the nucleation probability in the classical nucleation theory of phase transitions (*4*). In polymer physics, this question emerges in the problems of DNA melting (*5*, *6*), protein and RNA hairpin folding (*7*, *8*), polymer translocation through a small pore (*9*) and crystallization (*10*, *11*), polymer adsorption/desorption kinetics (*12*), and, related to it, cell adhesion (*13*, *14*). Estimating conditional splitting probabilities is also important to determine entropy production (*15*–*18*). Another important field of applications, to which we will refer through this paper, is given by competing (also known as parallel or concurrent) chemical (*19*), biochemical (*20*), or photochemical (*21*) reactions.

The key quantity characterizing generic competitive events is the splitting probability, i.e., the probability, for a random process, of realizing first a given event before several others could occur, and as such belongs to the class of first-passage observables. Most available theoretical methods to determine splitting probabilities are limited to one-dimensional (1D) Markovian processes (*22*, *23*). Recent advances have considered the extension to higher dimensions for Brownian random walks (*24*–*30*) and general Markovian processes (i.e., processes without memory) (*31*, *32*).

However, memory effects are essential in complex systems since they emerge as soon as the evolution of the random walker, or of the reaction coordinate, arises from interactions with other (possibly hidden) degrees of freedom. For example, the motion of a monomer in a macromolecule (*33*–*35*), or that of a particle in a crowded narrow channel (*36*), displays strong memory effects. Another well-known experimental example of a non-Markovian process (to be studied below) is the motion of a tracer bead in a viscoelastic solution (*37*–*40*), for which examples of mean square displacement (MSD) functions are shown on Fig. 1B, which clearly display several temporal regimes and strongly differ from Brownian motion, as expected in such complex fluids (*41*). The Gaussian nature of this process, as seen in Fig. 1C, together with the temporal nonlinearity of the MSD, ensures that the observed process is indeed non-Markovian (*42*).

However, a general theory to quantify the impact of memory effects on splitting probabilities is lacking. In the context of first-passage problems for non-Markovian processes, theoretical approaches have mainly been limited to the case of single targets (*43*–*51*). In the case of two targets, the prediction of splitting probabilities is limited to 1D processes, in a few specific examples (*52*, *53*) or for scale invariant processes using scaling arguments (*54*) or perturbative methods (*55*).

Here, we introduce a general nonperturbative formalism to predict the outcome of competitive events for the wide class of nonsmooth isotropic Gaussian processes with stationary increments (see below for definition), in the case of two targets. Strikingly, on the basis of a prototypical experimental reaction scheme with tracer beads in viscoelastic fluids, we provide direct experimental evidence of the impact of memory effects on competitive reactions (see Fig. 1D for illustration), in agreement with our theoretical predictions, while so far experimental observations of first-passage properties of non-Markovian processes have been limited to persistence exponents (*44*, *56*) or passage over barriers (*57*, *58*). In particular, our observations provide a direct experimental proof that the state of the system (constituted by the random walker and the additional degrees of freedom of its environment) at the first-passage event is not an equilibrium state. Our theory extends to dimensions higher than 1, providing a path toward the understanding of competitive diffusion-limited reactions in complex systems.

## RESULTS

### General formalism

We first consider a random walker of position x(t) evolving in continuous time t in a 1D space (the generalization to higher dimensions will be considered afterward). We assume that the random walk is symmetric (no privileged direction) and that the increments are stationary (no aging). The initial position is $x_{0}$. We also assume that the process x(t) is continuous (no jumps) and nonsmooth (with formally infinite velocity, as in the case of overdamped processes and in particular of Brownian motion), and that it has Gaussian statistics. With these hypotheses, the process is fully characterized by its average $\left(\langle x\left(t\right)\rangle =x_{0}\right)$ and the MSD function $\psi \left(t\right)=\langle {\left[x\left(t\right)-x_{0}\right]}^{2}\rangle$. Last, we assume that, at long times only, ψ(t) behaves as $\psi \left(t\right)≃\kappa \;t^{2H}$ with κ>0 and 0<H<1. The hypothesis H>0 ensures that the particle does not remain trapped around a given position, and the condition H<1 ensures that the correlation function of the increments decays at long times. The process is therefore assumed to be diffusive (H=1/2), subdiffusive (H<1/2), or superdiffusive (H>1/2) at long times. With these hypotheses, one describes a large class of non-Markovian random walks, and in particular diffusion of beads attached to macromolecules (*33*, *34*, *59*–*61*), or moving in viscoelastic fluids (*37*, *38*) or crowded narrow channels (*36*), etc. We stress that the above assumptions refer to the dynamics of the random walker in the absence of targets.

We now consider two perfectly absorbing targets at positions $x_{1}=0$ and $x_{2}=L$ (with $0<x_{0}<L$). The random walk ends whenever one of these two regions is reached, and we aim to calculate $\pi_{i}$, the probability that the target i∈{1;2} is reached first. In the single target problem (*43*, *45*, *51*), it was previously shown that a key quantity to predict first-passage statistics is the average trajectory after first contact, if the random walker were allowed to continue its motion. We thus introduce $\mu_{1}\left(t\right)$ and $\mu_{2}\left(t\right)$, the average trajectories at a time t after a first contact with targets 1 and 2, respectively. The following probabilistic argument enables one to understand why $\mu_{1}$ and $\mu_{2}$ are inherently linked to the splitting probabilities. At long times, the average of x(t) (without targets) is clearly $x_{0}$, but on the other hand, the average of x can be computed by partitioning over the first contacts with each of the targets, leading to

$$x_{0}=\underset{t\to \infty }{\text{lim}}\left[\pi_{1}\mu_{1}\left(t\right)\;+\;\pi_{2}\mu_{2}\left(t\right)\right]$$ (1)

Note that, for the (Markovian) Brownian motion, $\mu_{1}=0$ and $\mu_{2}=L$ so that the above argument leads to the well-known result $\pi_{2}=x_{0}/L$. In the more general case of non-Markovian processes, this equations is key to evaluate $\pi_{1}$ (and $\pi_{2}=1-\pi_{1}$), but requires the knowledge of $\mu_{1}$ and $\mu_{2}$. A self-consistent equation for $\mu_{1}$ and $\mu_{2}$ can be obtained by assuming that the statistics of trajectories after a first contact is Gaussian, with the same covariance as that of the original process (see section S1). These assumptions are well supported by simulations (fig. S1). The equations for $\mu_{1},\mu_{2}$ are, for i∈{1,2}

$$\begin{array}{c} 0=\sum_{j=1}^{2}\pi_{j}\int_{0}^{\infty }\mathrm{dt}\left(p\left(x_{i},t\right)\left(x_{0}-x_{i}\right)[1-M(t,\tau )]\right.\\ -q_{j}\left(x_{i},t\right)\{\mu_{j}\left(t+\tau \right)-x_{i}-\left[\mu_{j}\left(t\right)-x_{i}\right]M(t,\tau )\}) \end{array}$$ (2)

with

$$\begin{array}{c} M\left(t,\tau \right)=\left[\psi \left(t+\tau \right)+\psi \left(t\right)-\psi \left(\tau \right)\right]/\left[2\psi \left(t\right)\right]\\ q_{j}\left(x_{i},t\right)=\frac{e^{-\frac{{\left(x_{i}-\mu_{j}\left(t\right)\right)}^{2}}{2\psi \left(t\right)}}}{{\left[2\pi \psi \left(t\right)\right]}^{1/2}},p\left(x_{i},t\right)=\frac{e^{-\frac{{\left(x_{i}-x_{0}\right)}^{2}}{2\psi \left(t\right)}}}{{\left[2\pi \psi \left(t\right)\right]}^{1/2}} \end{array}$$ (3)

Here, $p\left(x_{i},t\right)$ is the value of the probability density function (PDF) of the initial process at the position $x_{i}$, while $q_{j}\left(x_{i},t\right)$ is the PDF of observing a particle at position $x_{i}$ at a time t after the first contact, given that the target j is hit first; note that these quantities are defined for the process in infinite space. Equation 2 expresses the fact that the conditional average of x(t+τ), given that $x\left(t\right)=x_{i}$, averaged over t, can also be calculated by partitioning over events where the target j is hit first in terms of averaged trajectories after the first contact; the function M comes from general expression of conditional averages for Gaussian processes (*62*). The above equation (Eq. 2) can be solved numerically to evaluate $\mu_{1}\left(t\right)$ and $\mu_{2}\left(t\right)$, and therefore $\pi_{1}$ and $\pi_{2}$ by using Eq. 1, for arbitrary ψ(t).

**Fig. 2.**
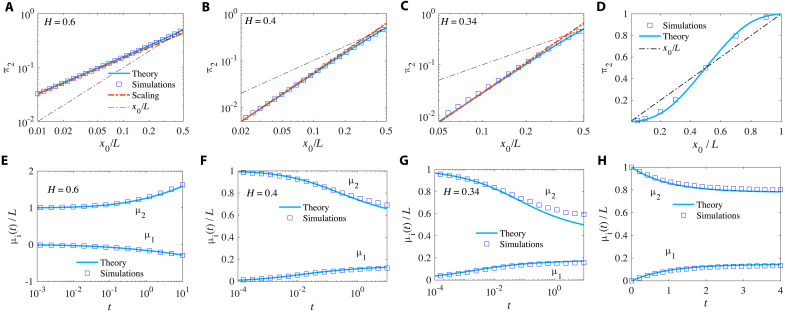
Splitting probabilities and mean trajectories after the first passage for 1D processes. (**A**) Splitting probability for a superdiffusive fBM with H=0.6. Symbols are simulation results obtained with the circulant matrix algorithm (*68*, *69*) (statistical error is smaller than symbol sizes). The blue continuous line is the theoretical prediction, obtained by numerically solving Eqs. 1 and 2. The red dashed line is the scaling (Eq. 4) with the prefactor $A_{H}$ predicted by our theory. The black dashed line is the formula $\pi_{2}=x_{0}/L$, obtained by setting $\mu_{1}=0$ and $\mu_{2}=L$, that overlooks non-Markovian effects. (**B** and **C**) Splitting probability for subdiffusive fBMs (H=0.4 and H=0.34), with the same color code as in (A). (**D**) Splitting probabilities for a nonscale invariant process with MSD $\psi \left(t\right)=t+B\left(1-e^{-t}\right)$ with B=10 (bidiffusive process), when the separation between the targets is L=20 (in dimensionless units), with the same color code as in (A) to (C). (**E** to **H**) Average trajectories $\mu_{i}\left(t\right)$ in the future of first-passage events as measured in simulations (symbols) and predicted by our theory (lines), for $x_{0}/L=$ 0.208, for the processes corresponding to (A) to (D), respectively. In (E) to (G), the time t is in units of ${\left(L/\sqrt{\kappa }\right)}^{1/H}$. In (A) to (C), only the range $x_{0}<L/2$ is shown. The values of $x_{0}>L/2$ can be deduced by symmetry. The same figure, with linear scales, is shown in fig. S2.

Several comments are in order. First, for weakly non-Markovian processes, i.e., when one can write $\psi \left(t\right)=2\mathrm{Dt}+{\varepsilon \psi }_{1}\left(t\right)$, with ε a small parameter, our theory provides exact results at order ε (section S5), and we obtain explicit formulas for the splitting probability at this order for any $\psi_{1}$, which agree with the result of (*55*) found with other methods in the particular case where $\psi \left(t\right)=\kappa \;t^{2H}$ with H→1/2. Second, we compare the predictions of our formalism to simulation results for several stochastic processes, including strongly non-Markovian processes. To this end, we consider two paradigmatic examples of Gaussian non-Markovian processes: (i) the fractional Brownian motion (fBM) with MSD $\psi \left(t\right)=\kappa \;t^{2H}$, which is scale invariant (at all times). This model displays long-range memory effects and appears in various fields; in particular, it can describe the dynamics of a monomer of an infinite polymer chain (*33*–*35*), or of a tagged particle in single-file diffusion (*36*). (ii) The “bi-diffusive” process, with MSD $\psi \left(t\right)=t+B\left(1-e^{-t}\right)$ (in dimensionless variables, with B>0). This process appears when x(t) is driven by the sum of a white noise and a colored one, with only one relaxation time, as in a Maxwell fluid (*63*). Numerical simulations of these processes show a quantitative agreement with our theoretical predictions for $\pi_{2}$ in all cases (see Fig. 2). Note that, in our calculations, we used $\pi_{2}={\left[\left(x_{0}-\mu_{1}\right)/\left(\mu_{2}-\mu_{1}\right)\right]}_{t\to \infty }$ and we checked that the convergence to the limit is fast enough to enable the efficient numerical determination of $\pi_{2}$ (even when $\mu_{1}$ and $\mu_{2}$ both diverge; see fig. S2). Of note, the Markovian prediction $\pi_{2}=x_{0}/L$ can either strongly underestimate (Fig. 2A) or overestimate (Fig. 2, B and C) $\pi_{2}$, with an obviously incorrect scaling behavior at small $x_{0}$, while our approach remains quantitative in this regime.

Third, we analytically examine the case of scale invariant processes, $\psi \left(t\right)=\kappa \;t^{2H}$, for which the dependence on the geometric parameters $x_{0}$ and L can be extracted. For $x_{0}\ll L$, we find

$$\pi_{2}\underset{x_{0}\ll L}{≃}A_{H}{\left(x_{0}/L\right)}^{1/H-1}$$ (4)

where the prefactor $A_{H}$ is determined below. Of note, this scaling behavior is consistent with that obtained from scaling arguments in (*54*) for 1D processes, and extended in (*64*) to higher dimensions, in agreement with earlier predictions for Markovian scale invariant processes (*31*). Our approach also provides the quantitative determination of the prefactor $A_{H}$, unknown so far. When $x_{0}/L\ll 1$, $\mu_{2}$ is not expected to depend on $x_{0}$, whereas $\mu_{1}$ varies at two time scales: the typical time to travel a distance $x_{0}$, equal to $\tau_{1}={\left(x_{0}/\sqrt{\kappa }\right)}^{1/H}$, and the time to travel a distance L, equal to $\tau_{2}={\left(L/\sqrt{\kappa }\right)}^{1/H}$. We find that the structure of $\mu_{1}$ in terms of matched asymptotic expansions is

$$\mu_{1}\left(t,x_{0}\right)≃\left\{\begin{array}{cc} x_{0}\;\mu_{\infty }\left(t/\tau_{1}\right) & \left(t\ll \tau_{2}\right)\\ x_{0}-x_{0}{\left(\frac{x_{0}}{L}\right)}^{\frac{1}{H}-2}\chi \left(t/\tau_{2}\right) & \left(t\gg \tau_{1}\right) \end{array}\right.$$ (5)

$$\mu_{2}\left(t,x_{0}\right)≃L\;m_{2}\left(t/\tau_{2}\right)$$ (6)

where $\mu_{\infty },m_{2}$, and χ are dimensionless scaling functions satisfying a set of equations identified in the Supplementary Materials (section S3, eqs. S26, S27, and S30). Using Eq. 1, it is clear that our formalism yields the scaling (Eq. 4) and provides the value of the prefactor $A_{H}={\left(\chi /m_{2}\right)}_{t\to \infty }$, in excellent agreement with simulation results (see Fig. 2) in the regime $x_{0}\ll L$.

### Experiments

We have experimentally measured first-passage events for non-Markovian processes by observing the motion of micrometer-sized beads in viscoelastic large polymer weight solutions (details on experiments can be found in Materials and Methods and section S4). The beads are far from the confining boundaries containing the polymer fluid, and we focus on their motions along the x axis, which were tracked by using optical microscopy. This type of experimental setup is standard in microrheology (*37*–*40*, *65*) but is usually used to measure viscoelastic parameters and not first-passage properties. The motion of the beads can be interpreted as obeying an overdamped generalized Langevin equation (GLE)

$$\int_{0}^{t}\mathrm{dt}\prime K\left(t-t\prime \right)\dot{x}\left(t\prime \right)=\xi \left(t\right)$$ (7)

where the Gaussian noise ξ(t) has vanishing average and satisfies $\langle \xi \left(t\right)\xi \;(t\prime )\;\rangle =k_{B}\mathrm{TK}\left(\;|t-t\prime \;|\;\right)$. The measured MSDs in Fig. 1C typically display two regimes: one long-time diffusive regime and one short-time regime where one observes apparent anomalous diffusion. This suggests that, to account for the observed trajectories, one can use a friction kernel of the form of

$$K\left(t\right)=\frac{\gamma_{0}\tau_{0}^{\alpha -1}e^{-t/\tau_{0}}}{\Gamma \left(1-\alpha \right)t^{\alpha }}$$ (8)

where $\tau_{0}$ is the relaxation time of the polymer solution, $\gamma_{0}=\int_{0}^{\infty }\mathrm{dtK}\left(t\right)$ is the long-time friction coefficient, and α is the subdiffusion exponent at small times. For this memory friction kernel, the MSD reads

$$\psi \left(t\right)=\frac{2k_{B}T\tau_{0}}{\gamma_{0}}f\left(t/\tau_{0}\right)$$ (9)

$$f\left(y\right)=\left[\left(y-\alpha +1\right)\gamma (\alpha ,y)+y^{\alpha }e^{-y}\right]/\Gamma \left(\alpha \right)$$ (10)

where $\gamma \left(\alpha ,y\right)=\int_{0}^{y}t^{\alpha -1}e^{-t}\mathrm{dt}$ is the lower incomplete gamma function. With this choice of kernel, the MSD displays a short-time anomalous diffusive regime $\psi \left(t\right)\propto t^{\alpha }$ and a long-time diffusive one, and $\tau_{0}$ is the crossover time between these regimes. The fits of experimental MSD curves show a good agreement, as seen on Fig. 1B, and support this choice of function for K. We checked that the stochastic process x(t) in the experiment is Gaussian (Fig. 1C) and unbiased (section S4), as it should be if it is a realization of the GLE (*7*), and as implicitly assumed in all microrheology experiments.

**Fig. 3.**
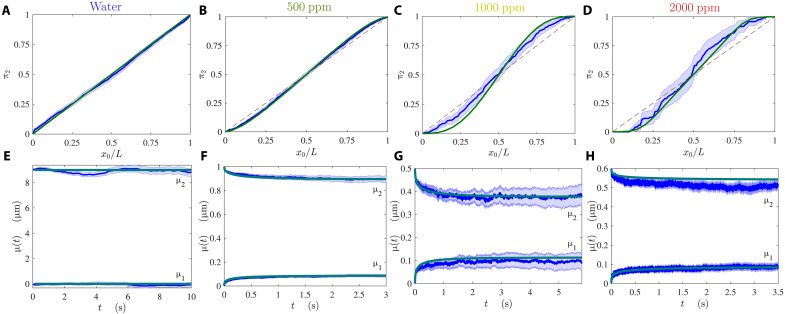
Experimental and theoretical splitting probabilities and mean trajectories after the first passage for a random walker in a viscoelastic fluid. (**A** to **D**) Values of the splitting probabilities measured in experiments (blue lines, surrounded by estimates of 95% error bars), compared with the prediction of our theory (green line). The polymer concentration is indicated on each graph. We also show the result for Markovian diffusion $\pi_{2}=x_{0}/L$ (red dashed line). (**E** to **H**) Average trajectories $\mu_{1}$ and $\mu_{2}$ after the first passage to targets 1 and 2, as measured in the experiments (blue lines) and predicted by the theory (green lines). Parameters: [(A) and (E)]: c=0 ppm (water) and L=9μm; [(B) and (F)]: c=500 ppm and L=1μm; [(C) and (G)]: c=1000 ppm and L=0.5μm; [(D) and (H)]: c=2000 ppm and L=0.6μm. In (E), $x_{0}=2\;\mu$m; in (F) to (H), $x_{0}=0.2\;\mu$m. Error bars indicate 95% confidence intervals and are calculated with the following number of recorded first-passage events: n=1001 for c=0 ppm, n=2771 for c=500 ppm, n=350 for c=1000 ppm, and n=116 for c=2000 ppm.

Next, we investigated the first-passage properties of a trajectory x(t) starting at $x\left(t_{0}\right)=x_{0}$: We measured the variable η defined as =1 if a fictitious target at position L is hit before the position 0. To obtain enough statistics, we considered that $x\left(t_{0}\right)$ and $x\left(t_{1}\right)$ could be used as independent starting positions if the time elapsed between $t_{1}$ and $t_{2}$ is larger than $2\tau_{0}$ (or 2 s in water). Then, $\pi_{2}\left(x_{0},L\right)$ was calculated as an ensemble average 〈η〉 over different starting positions on each trajectory for various bead trajectories. In our experiments, we made sure that the frame rate is large enough to consider that the typical distances traveled during each time step dt is much smaller than L. The results for $\pi_{2}$ are displayed on Fig. 3 (B to D). It is clear that for strongly viscoelastic solutions, for which α is the lowest, the curve $\pi_{2}\left(x_{0}\right)$ is very different from a straight line (which is the result for Markovian diffusion; see Fig. 3A), indicating that the probability to hit the closest target is increased by memory effects. This comes from the fact that, in polymer fluids, the motion of a tracer bead induces a delayed response of the surrounding polymer network, tending to bring it back to previously occupied positions, inducing a “denser” exploration of space and a larger probability to hit the closest targets. Furthermore, for all parameters, the experimental values of $\pi_{2}$ are in good agreement with our theoretical predictions obtained by solving Eqs. 1 and 2 by using the previously fitted MSD (Eq. 9).

In our experiments, we also measured the trajectories $\mu_{1}\left(t\right),\mu_{2}\left(t\right)$ followed by x(t) after the first-passage events, which are the hallmarks of non-Markovian effects in the theoretical approach above. These trajectories are displayed in Fig. 3 (F to H) where it is clearly seen that, on average, x(t) does not stay at x=0 or x=L after the targets have been reached. These trajectories $\mu_{1}$ and $\mu_{2}$ are in quantitative agreement with their predicted values. In our experiments, the motion of a bead with an equilibrium initial condition would be unbiased. In turn, our observation that $\mu_{i}\neq x_{i}$ indicates that the state of the polymer fluid upon a first-passage event at a target is not an equilibrium state. Physically, this comes from the fact that the fluid exerts a delayed response force tending to bring the bead back to its previously occupied positions, which, in our situation, are inside the interval [0,L]. This effect is, as expected, not present in solutions without polymers (see Fig. 3E). Our observations thus constitute a direct experimental proof showing unambiguously that the state of a system (constituted by the bead and the surrounding polymer fluid) upon a first-passage event is not an equilibrium one, which is crucial to understand first-passage properties.

### Extension to higher spatial dimensions (d>1)

Our theory can be generalized to higher dimensions, which is relevant to describe general competitive reactions. We denote the d-dimensional trajectory of the random walker by $r\left(t\right)=\left[x_{1}\left(t\right),x_{2}\left(t\right),\ldots x_{d}\left(t\right)\right]$. We assume the presence of two targets of finite radius a around locations $r_{1}$ and $r_{2}$, while $r_{0}$ is the starting position. The dynamics is assumed to take place in a confining volume so that the PDF of positions in confinement $p^{c}\left(r,t\right)$ reaches a stationary value $p_{s}^{c}\left(r\right)$ at large time (note that for d=1, the presence of confining walls, if beyond the targets, becomes irrelevant). We assume that, in the limit of large volume, far from the boundaries, all $x_{i}\left(t\right)$ satisfy the hypotheses used for the motion of x(t) in 1D. We also assume isotropy so that the coordinates $x_{i}\left(t\right)$ are independent. We show in section S6 that, in the large volume limit

$$\pi_{1}=1-\pi_{2}\underset{V\to \infty }{≃}\frac{h_{22}-h_{12}}{h_{22}+h_{11}-h_{21}-h_{12}}$$ (11)

where

$$h_{\mathrm{ij}}=\;\int_{0}^{\infty }\mathrm{dt}\;\left[q_{j}\left(r_{i},t\right)-p\left(r_{i},t\right)\right]$$ (12)

where $q_{j}\left(r,t\right)$ is the PDF of the position r at a time t after the first passage to target j, and p is the probability density of the initial process in unconfined space. Note that, in our formalism, in the large volume limit, the propagators appearing in Eq. 12 are evaluated by considering the dynamics in infinite space (without confining boundaries or targets). The above equations generalize similar equations for Markovian processes (*31*, *32*) to non-Markovian ones. Although the expressions are similar, the main difference from Markovian processes is that here the propagators have to be evaluated in the future of first-passage events, which can strongly differ from the dynamics in the future of a stationary state. In d=1, Eq. 11 is an alternative formula to estimate $\pi_{i}$, which, however, gives results that are indistinguishable from those obtained with Eq. 1 (see section S6 and fig. S4).

**Fig. 4.**
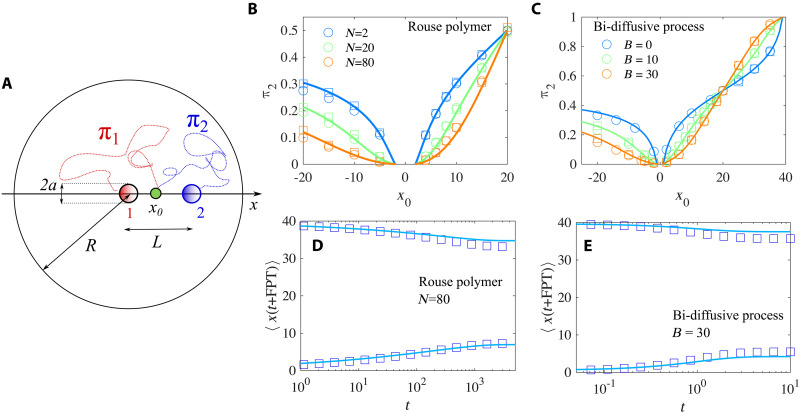
Splitting probabilities in two dimensions. (**A**) Sketch of the competitive event problem in two dimensions, when the confining volume is a disk of radius R centered about the first target, with L the distance between the targets and a their radii. Here, $x_{0}$ is located on the line joining the centers of the targets and the position of the first target is $x_{1}=0$. (**B**) Splitting probability when the random walker is the first monomer of bead-spring (Rouse) polymer chain of N monomers, whose dynamics obeys $\partial_{t}x_{i}=x_{i+1}-2x_{i}+x_{i-1}+f_{i}\left(t\right)$, with the prescription $x_{0}=x_{1}$ and $x_{N}=x_{N+1}$ at the ends, with $\langle f_{i}^{\alpha }\left(t\right)f_{j}^{\beta }\left(t\prime \right)\rangle =2\delta_{\alpha \beta }\delta_{\mathrm{ij}}\delta \left(t-t\prime \right)$, with i,j the monomers’ indexes and α,β the spatial coordinates. Symbols are simulation results for a=2, L=40, and N=2 (blue), N=20 (green), and N=80 (red); circles are results for R=80 and squares for R=160. Lines represent theoretical predictions (Eqs. 11 and 12). (**C**) Splitting probability for the bidiffusive process with MSD $\psi \left(t\right)=t+B\left(1-e^{-t}\right)$ for each coordinate. Symbols are simulation results for a=1, L=40, and B=0 (blue), B=10 (green), and B=30 (red), for R=90 (circles) and R=200 (squares). (**D** and **E**) Projection of the mean trajectory after the first passage time (FPT) on the *x* axis, when the target 1 is hit before (lower curves) or after (upper curves) the target 2 for (D) Rouse dynamics (with N=80, $x_{0}=15$, R=160, a=2, L=40) and (E) for the bidiffusive process (with B=30, $x_{0}=14$, R=90, a=1, L=40). Lines are theoretical predictions.

In d-dimensions, geometric effects are more difficult to take into account than in the 1D case, since it is necessary to evaluate in which direction the random walker moves after hitting a target. We address this problem by using the following approximations: (i) We work within a decoupling approximation so that the statistics of paths in the future of the first passage to target i is assumed to be the same as in the single target problem, and (ii) we assume that the paths in the future of a first contact with target i at hitting angle θ follow a Gaussian distribution, with mean $\mu_{i}\left(t\right)$ oriented along this angle θ, which itself has a distribution $\Pi \left(\theta \right)\propto e^{\alpha \text{cos}\left(\theta -\theta_{0}\right)}$. This form for Π(θ) is a minimal ansatz of positive 2π periodic function, which is in good agreement with simulations (fig. S6), and we have written self-consistent equations for $\mu_{i}\left(t\right)$ and α (see section S6), which provide $\pi_{2}$. Although the theory in d-dimensions involves more approximations than in the 1D case, the results shown in Fig. 4 demonstrate that our theory captures well the effects of memory on the splitting probabilities for competitive reactions in dimension higher than one.

## DISCUSSION

Here, we have presented a general theory that predicts the effect of memory on the outcome of competitive events, quantified by the splitting probability to reach one target before the other for nonsmooth isotropic Gaussian stochastic processes with stationary increments. Our theory is exact at first nontrivial order for weakly non-Markovian processes and, beyond this perturbative regime, in quantitative agreement with both numerical simulations and experiments where the realization of the random walk is the motion of a tracer bead in a viscoelastic fluid. For this class of processes, the effect of memory is to increase the probability to hit the closest target (with respect to the Markovian prediction). This effect is also clear by looking at the case of Gaussian subdiffusive processes. This effect is strongly different from the case where subdiffusion arises from random jumps with heavy-tailed distributed waiting times, since the distribution of waiting times does not influence splitting probabilities (*31*). Our experiments also unambiguously demonstrate that the state of the system (formed by the bead and the surrounding bath) at the first passage is not an equilibrium one being conditioned to the random walker x being at one of the targets, as seen from the biased dynamics after the first-passage events (while the initial process is not biased); this aspect is actually intrinsically linked to splitting probabilities (see Eq. 1). Our theory can be extended to cover the case of reactions in spatial dimension higher than one in the presence of a large confining volume, opening a path to the study of the impact of memory effects on competitive reactions in complex media.

## MATERIALS AND METHODS

### Preparation of polymer solutions, particle suspension, and tracking methods

Polyacrylamide [molecular weight (*M*_w_) = 18 × 10^6^ g/mol, from Polysciences] was dissolved in Millipore water (18.2 megohm·cm). Sodium chloride (NaCl; 10 mM; from Sigma-Aldrich) was added in the solution. The solution was then placed on a digital roller shaker (IKA Roller 6) for around 80 hours at a speed of 20 to 30 rpm at room temperature to dissolve the polymer completely. The solution was stored in a fridge at 4° to 6°C. To minimize the effect of solution aging, all solutions were used in this study within 1 month; we checked using rheological measurements that the solutions remained intact for such a period of time. Polystyrene particles from Invitrogen with diameter 1 μm were used in the experiments. Typically, 1.0 μl of original particle solution was added into a 2.5 to 3.0 ml of polymer solution. The particle suspension was then mixed by using the digital roller shaker for 10 hours at 20 to 30 rpm. Experiments were carried out under a darkfield inverted microscope (Zeiss AXIO Observer). The diluted particle suspension was sealed inside an adhesive incubation chamber (from Bio-Rad, 9 mm × 9 mm, 25 μl). The chamber was covered by a thin cover slide due to the limitation of the working distance of the darkfield condenser. The darkfield condenser was immersed in an optical oil over the microscope, and an objective (Olympus, SLMPLNx100) with magnification ×100 and numerical aperture 0.6 was used to visualize the particle motion. A motorized translation stage was used to capture microparticles from different areas. All videos were recorded by using a high-resolution camera (Hamamatsu, OrcaFlash 4.0 C11440). To minimize the memory effect of the polymer solution, different places were chosen to record the particle motion. All particles were tracked with TrackPy (based on Python) (*66*, *67*). Additional details for the choice of experimental parameters, the estimator of the MSD and its variance, and the check of the absence of global drift can be found in section S4.
