# Supplementary material for: Evidence and quantification of memory effects in competitive first-passage events
Source: Sci Adv. 2025 Mar 21;11(12):eadp2386. doi: 10.1126/sciadv.adp2386 (PMC11927618; doi:10.1126/sciadv.adp2386)
Supplement: Supplementary file 1 — Supplementary Text Figs. S1 to S6 Tables S1 and S2 [file sciadv.adp2386_sm.pdf]

Science Advances
AAAS

Supplementary Materials for

Evidence and quantification of memory effects in competitive first-passage events

Maxim Dolgushev *et al.*

Corresponding author: Olivier Bénichou, benichou@lptmc.jussieu.fr

Sci. Adv. **11**, eadp2386 (2025)
DOI: 10.1126/sciadv.adp2386

This PDF file includes:

Supplementary Text
Figs. S1 to S6
Tables S1 and S2

In the supplementary materials, we provide

- a detailed derivation of the formalism to calculate the splitting probabilities in $d = 1$ (Section I),
- a control of the approximations (Section II),
- an asymptotic analysis of the theory for scale invariant processes (fBM) (Section III),
- details on experimental methods (Section IV),
- the proof that the theory is exact at first order for weakly non-Markovian processes and the solution of the perturbation theory (Section V),
- the extension of the formalism to higher dimensions (Section VI).

I. FORMALISM FOR THE TWO TARGET PROBLEM IN ONE DIMENSION

A. Derivation of Eq. (1)

In this section, we show that

$$x_0 = \lim_{t\to\infty} [\pi_1 \mu_1(t) + \pi_2 \mu_2(t)], \tag{S1}$$

which is Eq. (1) in the main text. We recall that the stochastic process is Gaussian, unbiased, with MSD $\psi(t)$, starting at x_0, with stationary increments, so that its covariance function is (see *e.g.* (43))

$$\mathrm{cov}(x(t), x(t')) \equiv \sigma(t,t') = \frac{1}{2}\left[-\psi(|t-t'|) + \psi(t) + \psi(t')\right]. \tag{S2}$$

Let us first write the exact relation

$$x_0 = \int_0^t dt' F(t') \mathbb{E}(x(t)|\mathrm{FPT} = t') + \mathbb{E}(x(t)|\mathrm{FPT} > t) S(t), \tag{S3}$$

where $F(t)$ is the density of first passage times (to reach either target 1 or target 2), $S(t) = \int_t^\infty d\tau F(\tau)$ is the survival probability, $\mathbb{E}(x(t)|\mathrm{FPT} = t')$ is the average of $x(t)$ given that the first passage time (FPT) is equal to t', $\mathbb{E}(x(t)|\mathrm{FPT} > t)$ is the average of $x(t)$ given that the first passage is larger than t. We introduce the following "trick", for any $A > 0$ and any function $g(t,t')$:

$$\int_0^A dt \int_0^t dt'\; g(t,t') = \int_0^A dt' \int_{t'}^A dt\; g(t,t') = \int_0^A dt' \int_0^{A-t'} du\; g(t'+u, t') = \int_0^A du \int_0^{A-u} dt'\; g(t'+u,t'). \tag{S4}$$

Using this property, if we integrate (S3) over t between 0 and A, we obtain

$$\int_0^A dt\; x_0 = \int_0^A du \int_0^{A-u} dt'\; F(t') \mathbb{E}(x(t'+u)|\mathrm{FPT} = t') + \int_0^A dt\; \mathbb{E}(x(t)|\mathrm{FPT} > t) S(t). \tag{S5}$$

We consider the average trajectory $\mu(t)$ in the future of the FPT:

$$\mu(t) = \langle x(t + \mathrm{FPT})\rangle = \int_0^\infty dt' F(t') \mathbb{E}(x(t'+t)|\mathrm{FPT} = t'). \tag{S6}$$

Using (S5) and (S6) we obtain

$$\int_0^A dt\; [\mu(t) - x_0] = \int_0^A du \int_{A-u}^\infty dt'\; F(t') \mathbb{E}(x(t'+u)|\mathrm{FPT} = t') - \int_0^A dt\; \mathbb{E}(x(t)|\mathrm{FPT} > t) S(t) \equiv Q(A), \tag{S7}$$

where $Q(A)$ is defined by the above equation. We now wish to show that $Q(A)/A$ vanishes for large A. First, we set $v = A - u$:

$$Q(A) = \int_0^A dv \int_v^\infty dt' \; F(t')\mathbb{E}(x(t' + A - v)|\text{FPT} = t') - \int_0^A dt \; \mathbb{E}(x(t)|\text{FPT} > t)S(t), \tag{S8}$$

Next, when A goes to infinity at fixed t', v, the distribution of $x(t' + A|\text{FPT} = t')$ extends at most over a typical length $\sqrt{\kappa}A^H$ (found by dimensional analysis). Hence, the average distance $\mathbb{E}(x(t' + A)|\text{FPT} = t')$ travelled during the time A cannot be very large compared to this typical length $\sqrt{\kappa}A^H$, whatever the value of the first passage time t'. We thus argue that there exists a constant K_1 such that, for $A \to \infty$ (at fixed t', v), with $A \gg t'$,

$$|\mathbb{E}(x(t' + A - v)|\text{FPT} = t')| < K_1(A - v)^H < K_1 A^H. \tag{S9}$$

Hence, in the limit $A \to \infty$, we obtain

$$|\int_0^A dv \int_v^\infty dt' \; F(t')\mathbb{E}(x(t' + A - v)|\text{FPT} = t')| < \int_0^A dv \int_v^\infty dt' \; F(t')K_1 A^H. \tag{S10}$$

Note that, although in the above integral the upper bound for t' is formally equal to infinity, in practice only finite values of t' matter since $F(t')$ decays very fast at times larger than $(L^2/\kappa)^{1/(2H)}$ (beyond which the probability of having missed the targets becomes exponentially small). The same remark holds for the variable v. Hence, the limit $A \to \infty$ of Q can be evaluated by using $A \gg t', v$ Furthermore, the absolute value of $x(t)$, given that no boundaries have been reached before t, is necessarily less that L, so that $|\mathbb{E}(x(t)|\text{FPT} > t)| < L$. Using these arguments, we find

$$|Q(A)| < \int_0^A dv S(v)(K_1 A^H + L) < (K_1 A^H + L) \int_0^\infty dv S(v). \tag{S11}$$

Note that $\langle T \rangle = \int_0^\infty dv S(v)$ is finite in our case, because for times larger than $(L^2/\kappa)^{1/(2H)}$ the random walker is almost sure to have reached one of the two targets. The above expression tells us that $Q(A)$ is at most of order A^H for large A. Comparing with (S7), this means that, for large t, $\mu(t) - x_0$ is at most of order $1/t^{1-H}$. Hence, for $H < 1$, $\mu(t) - x_0$ vanishes at large times. Since $\mu = \pi_1\mu_1 + \pi_2\mu_2$, we obtain the result (S1), which is Eq. (1) of the main text.

B. Self-consistent equations for $\mu_1(t)$ and $\mu_2(t)$ [Derivation of Eq. (2)]

Let us consider the equation

$$p(0, t; y, t + \tau) = \int_0^t dt' F(t')p(0, t; y, t + \tau|\text{FPT} = t'), \tag{S12}$$

which is exact for continuous non-smooth processes. $p(0, t; y, t+\tau)$ is the joint probability density of observing $x(t) = 0$ and $x(t+\tau) = y$ (in the absence of any target), with $\tau > 0$ and $t > 0$. Next, $p(0, t; y, t+\tau|\text{FPT} = t')$ is the probability density of observing $x(t) = 0$ and $x(t+\tau) = y$ given that the FPT (to reach any of the two targets) is t', if the random walker is allowed to continue its motion after the first passage. Using Eq. (S4), we obtain

$$\int_0^A dt \; p(0, t; y, t + \tau) = \int_0^A du \int_0^{A-u} dt' F(t')p(0, t' + u; y, t' + u + \tau|\text{FPT} = t'). \tag{S13}$$

Now, we introduce the joint probability to observe the position x at time t after the FPT and y at time $t + \tau$ after the FPT:

$$p_\pi(x, t; y, t + \tau) = \int_0^\infty dt' \; F(t') \; p(x, t' + u; y, t' + u + \tau|\text{FPT} = t'). \tag{S14}$$

Using the trick $\int_0^{A-u}(...) = \int_0^\infty(...) - \int_{A-u}^\infty(...)$ and the above definition, Eq. (S13) becomes

$$\int_0^A dt \; [p_\pi(0, t; y, t + \tau) - p(0, t; y, t + \tau)] = \int_0^A du \int_{A-u}^\infty dt' F(t')p(0, t' + u; y, t' + u + \tau|\text{FPT} = t'). \tag{S15}$$

We multiply the above equation by y, write $p_\pi(0,t;y,t+\tau)=\sum_{j=1}^2 \pi_j q_j(0,t;y,t+\tau)$, and integrate over y to obtain

$$\int_0^A dt\,\left[\sum_{j=1}^2 \pi_j q_j(0,t)\mathbb{E}_{\pi_j}(x(t+\tau)|x(t)=0)-p(0,t)\mathbb{E}(x(t+\tau)|x(t)=0)\right]=$$
$$\int_0^A du\int_{A-u}^\infty dt'F(t')p(0,t'+u|\mathrm{FPT}=t')\mathbb{E}(x(t'+u+\tau)|x(t'+u)=0;\mathrm{FPT}=t')\equiv R(A), \tag{S16}$$

where $R(A)$ is defined by the above equation, $\mathbb{E}_{\pi_j}(x(t+\tau)|x(t)=0)$ is the conditional average of $x(t+\tau+\mathrm{FPT})$ given that target j is reached first, and that $x(t+\mathrm{FPT})=0$. We wish now to show that $R(A)\to 0$ for $A\to\infty$. First, setting $u=A-v$ leads to:

$$R(A)=\int_0^A dv\int_v^\infty dt'F(t')p(0,t'+A-v|\mathrm{FPT}=t')\mathbb{E}(x(t'+A-v+\tau)|x(t'+A-v)=0;\mathrm{FPT}=t'). \tag{S17}$$

For large times, we argue that $p_\pi(0,t)\sim K_0/t^H$ for some $K_0>0$, since the distribution of positions extends over a length t^H. Hence,

$$p(0,t'+A-v|\mathrm{FPT}=t')\underset{A\to\infty}{\sim}\frac{K_0}{A^H}. \tag{S18}$$

Next, we argue that there exists a function $h(\tau)$ so that

$$|\mathbb{E}(x(\tau+t'+A-v)|x(t'+A-v)=0;\mathrm{FPT}=t')|<h(\tau), \tag{S19}$$

this is again related to the argument that the particle cannot travel an infinite distance during the time interval τ, whatever the conditioning on the past is. With these arguments, we obtain

$$|R(A)|<K_0\int_0^A dv\int_v^\infty dt'F(t')\frac{1}{A^H}h(\tau)\simeq\frac{1}{A^H}\int_0^\infty dvS(v)h(\tau)\qquad (A\to\infty). \tag{S20}$$

We conclude that $R(A)$ vanishes for large A. Therefore, taking the limit $A\to\infty$ in Eq. (S16) leads to

$$\mathcal{H}_1(\tau)\equiv\int_0^\infty dt\,\left\{\pi_1 q_1(0,t)\left[\mu_1(t+\tau)-\mu_1(t)\frac{\sigma(t+\tau,t)}{\sigma(t,t)}\right]+\pi_2 q_2(0,t)\left[\mu_2(t+\tau)-\mu_2(t)\frac{\sigma(t+\tau,t)}{\sigma(t,t)}\right]\right.$$
$$\left.-\,p(0,t)\left[x_0-x_0\frac{\sigma(t+\tau,t)}{\sigma(t,t)}\right]\right\}=0, \tag{S21}$$

where we have assumed that the stochastic process after hitting a target is Gaussian with the stationary covariance approximation, and we have used formulas for conditional averages of Gaussian variables, see *e.g.* (62):

$$\mathbb{E}(X|Y=y)=\mathbb{E}(X)-[\mathbb{E}(Y)-y]\frac{\mathrm{cov}(X,Y)}{\mathrm{var}(Y)}. \tag{S22}$$

Note that, since the process $x(t)$ has stationary increments, the covariance $\sigma(t,t')=\mathrm{cov}(x(t),x(t'))$ is given by Eq. (S2). Finally, with the same reasoning one obtains the equation related to the second target:

$$\mathcal{H}_2(\tau)\equiv\int_0^\infty dt\,\left\{\pi_1 q_1(L,t)\left\{\mu_1(t+\tau)-[\mu_1(t)-L]\frac{\sigma(t+\tau,t)}{\sigma(t,t)}\right\}+\pi_2 q_2(L,t)\left\{\mu_2(t+\tau)-[\mu_2(t)-L]\frac{\sigma(t+\tau,t)}{\sigma(t,t)}\right\}\right.$$
$$\left.-\,p(L,t)\left[x_0-(x_0-L)\frac{\sigma(t+\tau,t)}{\sigma(t,t)}\right]\right\}=0. \tag{S23}$$

Eqs. (S21) and (S23) are equivalent to Eq. (2) of the main text.

II. VALIDITY CONTROL OF THE APPROXIMATIONS OF THE THEORY

The theory presented in Appendix I relies on two assumptions: (1) the process in the future of the first passage to a target $i \in \{1, 2\}$ can be described as a Gaussian process, and (2) the covariance of the future of the first passage is approximated by the covariance of the original process. The validity of these assumptions is checked on Fig. S1. As a direct test of the theory, we also show the splitting probabilities for the fractional Brownian motion in linear scales, see Fig. S2**A-C**. Finally, on Fig. S2 **D-E**, as a direct test of Eq. (1), we show the quantity $(x_0 - \mu_1)/(\mu_2 - \mu_1)$, enabling one to check that it converges to π_2 for large times.

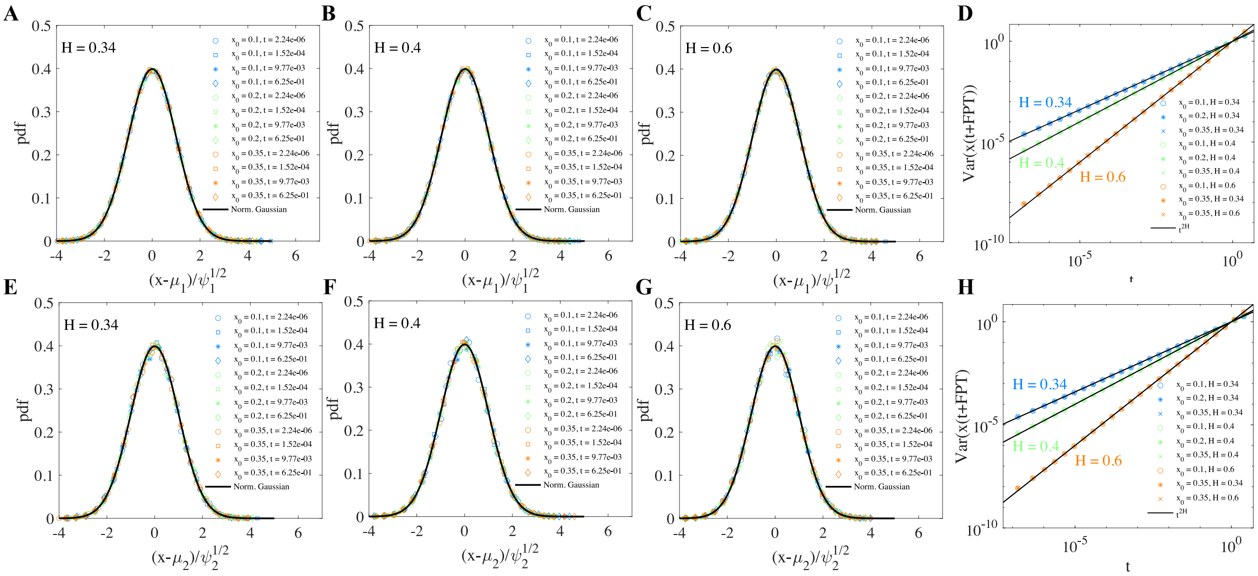

FIG. S1. **Numerical check of the hypotheses of the theory. A, B, C**: normalized histograms of $x(t + \text{FPT})$ given that target 1 is reached first. The stochastic process has mean square displacement $\psi(t) = t^{2H}$, with **A** $H = 0.34$, **B** $H = 0.4$, **C** $H = 0.6$ and the targets are at $x_1 = 0$ and $x_2 = L = 1$. The time step is $\Delta t = 1.5 \times 10^{-7}$. Symbols are the results for various values of t and x_0 indicated in legend. The black line is a normalized Gaussian. **D**: variance $\psi_1(t) = \text{var}[x(t + \text{FPT})]$ given that target 1 is reached first. Symbols are simulation results for various x_0, the black line represents $\psi_1(t) = t^{2H} = \psi(t)$. **E, F, G, H**: same figures when target 2 is reached first. Note that for the lowest values of x_0, the amount of recorded events is less than for target 1, explaining the higher dispersion of the data.

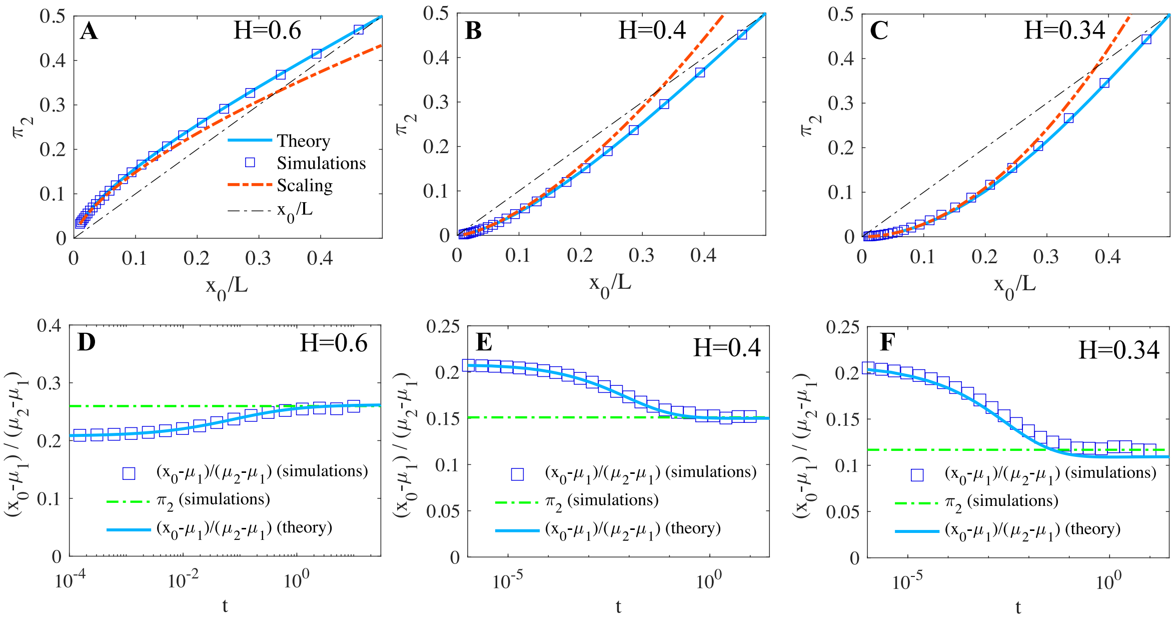

FIG. S2. **Splitting probabilities and check of the convergence of $(x_0 - \mu_1)/(\mu_2 - \mu_1)$ to π_2 at large times for the fractional Brownian motion.** Upper subfigures show the curves π_2 as a function of x_0, for **A**: $H = 0.6$, **B**: $H = 0.4$, **C**: $H = 0.34$. Symbols: simulations, blue line: theory, red dashed line: scaling (S25) with A_H as given in table S1. Black dot-dashed line: pseudo-Markovian estimate obtained with $\mu_i = x_i$. **A-C** are the same figures as in Fig. 2**A-C**, but in linear scales. Lower subfigures show $(x_0 - \mu_1)/(\mu_2 - \mu_1)$ as a function of time, for **D**: $H = 0.6$, **E**: $H = 0.4$, **F**: $H = 0.34$. Symbols: simulations, blue line: theory, green dot-dashed line: value of π_2 in simulations. Here, $x_0/L = 0.208$, and t is in units of $(L^2/\kappa)^{1/(2H)}$.

III. ASYMPTOTIC BEHAVIOR OF π_2 FOR SCALE INVARIANT PROCESSES [DERIVATION OF EQ. (4)].

Here, we consider the case $\psi(t) = \kappa\, t^{2H}$ at all times (so that, in the absence of target, $x(t)$ is a fBM). Without loss of generality, we chose the units of length and time so that $L = \kappa = 1$. We look for the value of π_2 for small x_0. We start with the following ansatz for the structure of the solution:

$$\mu_1(t, x_0) \simeq \begin{cases} x_0\ \mu_\infty(t/x_0^{1/H}) & t \ll 1, \\ x_0 - x_0^{1/H-1}\ \chi(t) & t \gg x_0^{1/H} \end{cases}, \qquad \mu_2(t, x_0) \simeq m_2(t), \tag{S24}$$

where μ_∞, χ and m_2 are scaling functions. This ansatz is justified by the fact that, for small x_0 it is clear that the time to reach the closest boundary must play a role, so that μ_1 must vary at the scale $x_0^{1/H}$. It must also vary at the second relevant time scale of the problem, i.e. the time to travel a distance L, which in our case is 1. As shown below, μ_∞ will be the reactive trajectory for the single target problem. Furthermore, the $x_0^{1/H-1}$ multiplicative factor of χ is imposed by the fact that $\mu_\infty(t) \simeq 1 - B_\infty/t^{1-2H}$, so that the small time and large time solutions coincide when $\chi(t) \underset{t\to 0}{\simeq} B_\infty/t^{1-2H}$. Finally, it is natural to assume that μ_2 does not vary at the scale $x_0^{1/H}$. The ansatz (S24) will be justified by the fact that one can identify the equations for χ, m_2 and μ_∞. The equation (S1) for π_1 and π_2 leads to

$$1 - \pi_1 = \pi_2 = A_H\ x_0^{1/H-1}, \qquad A_H = \lim_{t\to\infty} \frac{\chi(t)}{m_2(t)}, \tag{S25}$$

and thus the prefactor A_H of the scaling law for the splitting probability can be estimated from the values of m_2 and χ at infinity.

The equation for μ_∞ is obtained by estimating $\mathcal{H}_1(\tau)$ [defined in Eq. (S21)] for $\tau = x_0^{1/H}\overline{\tau}$, at fixed $\overline{\tau}$ in the limit

H	B_∞	A_H
0.4	0.75	1.75
0.34	0.49	2.5
0.6	1.05	0.69

TABLE S1. Values of B_∞ (calculated in (43)) and A_H, obtained numerically by solving (S27) and (S30).

of small x_0, which leads to

$$\int_0^\infty \frac{dt}{t^H}\left\{e^{-\mu_\infty^2(t)/2t^{2H}}\left[\mu_\infty(t+\overline{\tau})-\mu_\infty(t)\frac{t^{2H}+(t+\overline{\tau})^{2H}-\overline{\tau}^{2H}}{2t^{2H}}\right]-e^{-1/2t^{2H}}\left[1-\frac{t^{2H}+(t+\overline{\tau})^{2H}-\overline{\tau}^{2H}}{2t^{2H}}\right]\right\}=0, \tag{S26}$$

which is the equation for the single target problem (43) (as expected). Let us identify the equation for χ. With the above scaling ansatz (S24), we estimate that $\mathcal{H}_1(\tau)$ reads in the small x_0 limit (at fixed τ):

$$\mathcal{H}_1(\tau)\simeq -x_0^{1/H-1}\int_0^\infty\frac{dt}{t^H}\left\{\left[\chi(t+\tau)-\chi(t)M_H(t,\tau)\right]-A_He^{-m_2^2(t)/(2t^{2H})}\left[m_2(t+\tau)-m_2(t)M_H(t,\tau)\right]\right\}=0, \tag{S27}$$

where

$$M_H(t,\tau)=\frac{(t+\tau)^{2H}+t^{2H}-\tau^{2H}}{2t^{2H}}. \tag{S28}$$

Eq. (S27) provides an equation for χ and m_2. Next, we note that

$$e^{-\frac{[1-\mu_1(t)]^2}{2\psi}}\simeq e^{-\frac{\left[1-x_0+x_0^{1/H-1}\chi(t)\right]^2}{2t^{2H}}}\simeq e^{-1/(2t^{2H})}\left(1+\frac{x_0}{t^{2H}}-\chi(t)\frac{x_0^{1/H-1}}{t^{2H}}+...\right), \qquad (x_0\to 0). \tag{S29}$$

Hence, collecting the terms of order $x_0^{1/H-1}$ in $\mathcal{H}_2(\tau)$ [defined in Eq. (S23)], we obtain

$$\mathcal{H}_2(\tau)\simeq x_0^{1/H-1}\int_0^\infty\frac{dt}{t^H}\left\{\left[-\chi(t+\tau)+\chi(t)M_H(t,\tau)\right]e^{-1/(2t^{2H})}-\left(A_H+\chi(t)\frac{x_0^{1/H-1}}{t^{2H}}\right)[-1+M_H(t,\tau)]e^{-1/(2t^{2H})}\right.$$
$$\left.+A_He^{-[1-m_2(t)]^2/(2t^{2H})}\left[m_2(t+\tau)-1-[m_2(t)-1]M_H(t,\tau)\right]\right\}=0. \tag{S30}$$

Eqs. (S27) and (S30) form a system of two equations for χ and m_2 which we can solve, with the advantage that there are no parameters left (apart from H). We also note that we have to look for solutions with B_∞ as input, obtained by solving the equation for μ_∞, as in (43). Some results for A_H obtained by numerically solving Eqs. (S25), (S27) and (S30) are reported in table S1.

IV. DETAILS ON EXPERIMENTAL ANALYSIS

Estimation of the MSD and check of the no-drift hypothesis

To estimate the MSD $\psi(t)$ of the tracer particle, we used the following estimator, known as the time-averaged MSD, and defined as

$$\delta^2(\tau,T)=\frac{1}{T-\tau}\int_0^{T-\tau}dt\;[x(t+\tau)-x(t)]^2, \tag{S31}$$

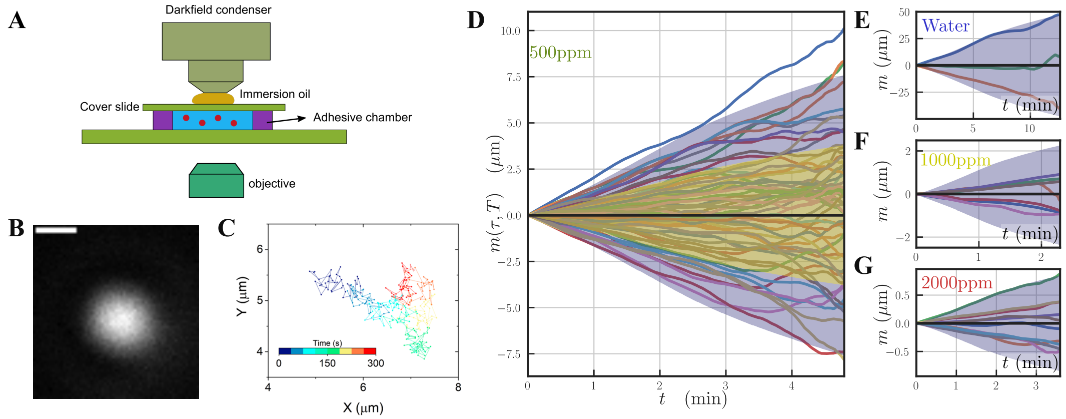

FIG. S3. **Tracking of particles in viscoelastic fluids.** **A** Schematic of darkfield experiment setup. The darkfield condenser is immersed in the optical oil. The thickness of the sample is about 30 μm sealed inside a slide incubation chamber (from Bio-Rad). **B** Example of image of a particle diffusing in the polymer solution, captured using dark field microscopy. **C** Example of trajectory of a particle diffusing in 2000 ppm polymer solution. The movie was recorded at 250 frame per second (fps) using an objective magnification 100×. Movie duration is about 5min. For clarity, a 1fps trajectory is plotted on the figure. **D, E, F, G** Check of the no-drift hypothesis: we represent the time averaged drift $m(t,\tau)$ defined in Eq. (S33). The purple conical regions are $\pm 2\sqrt{\text{var}(m(t,\tau))}$, which should contain 95% of the observations $m(t,\tau)$ in the absence of any drift. In **D**, the yellow conical region is $\sqrt{\text{var}(m(t,\tau))}$

c (ppm)	$\alpha/2$	γ_0/k_BT (s/μm^2)	τ_0 (s)
0 (water)	-	2.70±0.15	-
500	0.375± 0.005	40±2	1.5±0.2
1000	0.275±0.01	218±12	2.9±0.5
2000	0.175±0.01	(2.42±0.15)×10^3	7.0± 1

TABLE S2. Parameters of the MSD for the different polymer solutions, obtained by fitting the experimental MSDs with the functional form of Eqs. (9) and (10) in the main text. Uncertainties indicate values for which the fit becomes unsatisfactory, given the statistical uncertainties. Since no subdiffusive regime could be observed in the experiments for water, no attempt was made to measure values for τ_0 and α in that case.

where one assumes that a trajectory is observed during a time T. Obviously, $\langle \delta^2(\tau,T)\rangle = \psi(\tau)$, and the variance of δ^2 can be calculated by assuming that $x(t)$ is a Gaussian process (which is suggested by the experimentally observed histograms) with stationary increments. In this case,

$$\text{Var}(\delta^2(\tau,T)) = \int_0^{T-\tau} dz \frac{T-\tau-z}{(T-\tau)^2}\Big[\psi(z+\tau)+\psi(|z-\tau|)-2\psi(z)\Big]^2. \tag{S32}$$

This formula enables us to estimate the precision on the measurement of ψ to $\pm 2\sqrt{\text{Var}(\delta^2)/N_{\text{traj}}}$, with N_{traj} the number of independent observed trajectories. We then fit the functional form of the MSD using the equations (9) and (10) in the main text. The fitting parameters are indicated in table S2.

To determine if the deviations of $x(t)$ with respect to x_0 in each trajectory are related to a background drift flow, we estimated the time-averaged increments:

$$m(\tau,T) = \frac{1}{T-\tau}\int_0^{T-\tau} dt\ [x(t+\tau)-x(t)]. \tag{S33}$$

Under the hypothesis that x satisfies the GLE equation with no drift, $\langle m(\tau,T)\rangle = 0$ and its variance is

$$\langle m^2(t,\tau)\rangle = \int_0^{T-\tau} dz \frac{T-\tau-z}{(T-\tau)^2}\Big[\psi(z+\tau)+\psi(|z-\tau|)-2\psi(z)\Big]. \tag{S34}$$

In all our experiments, about 95% of the observed values of $m(\tau)$ remained in the range $\pm 2\langle m^2\rangle^{1/2}$ [calculated with

the above formula, see Fig. S3(d)-(f)], suggesting that there is no need to assume the existence of a drift to explain the data.

Choice of parameters

The choice of the parameters of the experiments is made to ensure that two conditions are satisfied. First, the displacement Δx between two frames has to be small compared to L, so that a first passage event between two frames is not missed in the analysis. Since $\Delta x \simeq \sqrt{\kappa_0}(\Delta t)^{\alpha/2}$ for small times, where $\kappa_0 = 2\tau_0^{1-\alpha} k_B T/(\gamma_0 \Gamma(1+\alpha))$ is the transport coefficient at small times, this condition writes

$$\frac{L}{\Delta x} = \frac{L}{\sqrt{\kappa_0}(\Delta t)^{\alpha/2}} \gg 1. \tag{S35}$$

We chose L to be the largest possible that remains in the range where the MSD is not linear, hence at the cross-over between the subdiffusive and the diffusive regime. Next, the memory of the camera limits the number of images that one can acquire during one experiment. For example, at our spatial resolution, the camera can take movies of about 64,000 images. Taking 10 movies (which represents 640GB of data), one can record about $n_0 \simeq 640,000$ images. If we consider that one can use initial conditions separated by $2\tau_0$ as independent initial conditions, we estimate the number of first passage events potentially observable as

$$N_{\rm events} \simeq \frac{(\Delta t)\times n_0}{2\tau_0} \gg 1 \tag{S36}$$

The temporal resolution Δt has to be chosen so that both conditions (S35) and (S36) are satisfied at the same time. For example, for a polymer solution concentrated at 1000 ppm, with $L = 0.5\mu m$, with a frame rate of 245 frames per seconds, we obtain $L/\Delta x \simeq 7$ and $N_{\rm events} \simeq 400$, which is the order of magnitudes of the number of events available for 640 GB of data. In our analysis we could actually observe about 500 first passage events without the bead leaving the frame or getting close to cell surfaces. Hence, a large amount of data was used to get enough statistics and observe with enough precision non-Markovian effects in the splitting probabilities.

V. FIRST ORDER PERTURBATION THEORY AROUND BROWNIAN MOTION ($d = 1$)

Here, we show that our theory is exact at first order for weakly non-Markovian processes. Our strategy consists in identifying an exact equation defining the distribution of paths after the first passage, and then checking that, with our Gaussian approximation and the stationary covariance hypothesis, this general equation is satisfied at first order. Next, we give the explicit solution for π_2 at this order, and compare with results of the literature.

A. Exactness of the theory at first order

Let us consider a set of times $\tau_1, ..., \tau_n$ and a set of positions $\{y_1, ..., y_N\}$. We may write the following equation for the probability density of observing the positions y_i at times $t + \tau_i$:

$$\begin{aligned} p(x_i, t; y_1, t+\tau_1; ...; y_N, t+\tau_N) = \int_0^t \mathrm{d}\tau' [F_1(\tau') p(x_i, t; y_1, t+\tau_1; ...; y_N, t+\tau_N | 1, \tau') \\ + F_2(\tau') p(x_i, t; y_1, t+\tau_1; ...; y_N, t+\tau_N | 2, \tau')], \end{aligned} \tag{S37}$$

where $p(x_i, t; y_1, t+\tau_1; ...; y_N, t+\tau_N | j, \tau')$ is the probability density of observing x_i at t and y_k at all $t + \tau_k$, given that target j was reached first at τ. Using exactly the same arguments as in Section I B, this equation leads to

$$\sum_{j=1}^{2} \pi_j \int_0^\infty \mathrm{d}t \, [q_j(y_1, t+\tau_1; ..., y_N, t+\tau_N | x_i, t) q_j(x_i, t) - p(y_1, t+\tau_1; ..., y_N, t+\tau_N |; x_i, t) p(x_i, t)] = 0. \tag{S38}$$

Note that for $N=0$ we obtain:

$$\sum_{j=1}^{2}\pi_j\int_0^\infty \mathrm{d}t\,[q_j(x_i,t)-p(x_i,t)]=0. \tag{S39}$$

Formally Eq. (S38) can be interpreted, in the continuous limit with $N\to\infty$ as

$$\sum_{j=1}^{2}\pi_j\int_0^\infty \mathrm{d}t\,[q_j([y(\tau)],t|x_i,t)q_j(x_i,t)-p([y(\tau)],t|x_i,t)p(x_i,t)]=0, \tag{S40}$$

for all continuous paths $[y_i(\tau)]$, satisfying $y_i(0)=x_i$, where $p([y_i(\tau)];t|x_0)$ is the joint probability density to follow this path after the time t, i.e. the probability that $x(t+\tau)=y_i(\tau)$ for all τ. Similarly, $q_j([y_i(\tau)],t|x_i,t)$ is the joint probability density that $x(t+\mathrm{FPT}+\tau)=y(\tau)$, for all $\tau>0$, given that target j was reached first, and that $x(t)=x_i$. Note that the condition $x(t)=x_i$ can be replaced by the condition that $y(0)=x_i$, and using (S39) one can write

$$\begin{aligned}\mathcal{G}_i([y])\equiv\sum_{j=1}^{2}\pi_j\int_0^\infty \mathrm{d}t\,\{&[q_j([y(\tau)],t|y(0)=x_i)-p_s([y(\tau)]|y(0)=x_i)]q_j(x_i,t)\\ &-p(([y(\tau)],t|y(0)=x_i)-p_s([y(\tau)]|y(0)=x_i)]p(x_i,t)\}=0,\end{aligned} \tag{S41}$$

where the functional $\mathcal{G}_i([y])$ is defined by the above equation for all paths $y(\tau)$, and $p_s([y(\tau)]|y(0)=x_i)=\lim_{t\to\infty}p([y(\tau)],t|y(0)=x_i)$ is the stationary probability to follow a the path $[y]$ given that it starts at x_i. The process corresponding to p_s is a Gaussian process of mean x_i and covariance $\sigma(t,t')$. The equation $\mathcal{G}_i([y])=0$, together with Eq. (S39) may thus be seen as a system of equation defining the distribution of paths after the first passage to one of the targets, and the splitting probabilities. Requiring that $\mathcal{G}_i([y])=0$ for all paths $[y(\tau)]$ is equivalent to requiring that the following functional vanishes for all functions $[k(\tau)]$:

$$\mathcal{F}_i([k])\equiv\int\mathcal{D}[y]e^{\mathrm{i}\int_0^\infty \mathrm{d}\tau k(\tau)y(\tau)}\mathcal{G}_i([y]). \tag{S42}$$

In the case that the paths after the FPT are Gaussian distributed, with mean $\mu_j(\tau)$ and covariance $\gamma_j(\tau,\tau')$ if target j is reached first, we can evaluate this functional:

$$\begin{aligned}\mathcal{F}_i([k])=&\int_0^\infty \mathrm{d}t\sum_{j=1}^{2}\pi_j\\ &\Big\{q_j(x_i,t)\Big[e^{\mathrm{i}\int_0^\infty \mathrm{d}\tau k(\tau)A_i^{\pi_j}(t,\tau)-\frac{1}{2}\int_0^\infty \mathrm{d}\tau\int_0^\infty \mathrm{d}\tau' k(\tau)k(\tau')B_i^{\pi_j}(t,\tau,\tau')}-e^{\mathrm{i}\int_0^\infty \mathrm{d}\tau k(\tau)x_i-\frac{1}{2}\int_0^\infty \mathrm{d}\tau\int_0^\infty \mathrm{d}\tau' k(\tau)k(\tau')\sigma(\tau,\tau')}\Big]\\ &-p(x_i,t)\Big[e^{\mathrm{i}\int_0^\infty \mathrm{d}\tau k(\tau)A_i(t,\tau)-\frac{1}{2}\int_0^\infty \mathrm{d}\tau\int_0^\infty \mathrm{d}\tau' k(\tau)k(\tau')B_i(t,\tau,\tau')}-e^{\mathrm{i}\int_0^\infty \mathrm{d}\tau k(\tau)x_i-\frac{1}{2}\int_0^\infty \mathrm{d}\tau\int_0^\infty \mathrm{d}\tau' k(\tau)k(\tau')\sigma(\tau,\tau')}\Big]\Big\},\end{aligned}$$

with

$$\begin{aligned}A_i^{\pi_j}(t,\tau)&=\mu_j(t+\tau)-[\mu_j(t)-x_i]\frac{\gamma_j(t+\tau,t)}{\gamma_j(t,t)}, & A_i(t,\tau)&=x_0-[x_0-x_i]\frac{\sigma(t+\tau,t)}{\sigma(t,t)},\\ B_i^{\pi_j}(t,\tau,\tau')&=\gamma_j(t+\tau,t+\tau')-\frac{\gamma_j(t+\tau,t)\gamma_j(t,t+\tau')}{\gamma_j(t,t)}, & B_i(t,\tau,\tau')&=\sigma(t+\tau,t+\tau')-\frac{\sigma(t+\tau,t)\sigma(t,t+\tau')}{\sigma(t,t)},\\ q_j(x_i,t)&=\frac{1}{\sqrt{2\pi\gamma_j(t,t)}}\exp\left[-\frac{[x_i-\mu_j(t)]^2}{2\gamma_j(t,t)}\right], & p(x_i,t)&=\frac{1}{\sqrt{2\pi\sigma(t,t)}}\exp\left[-\frac{[x_i-x_0]^2}{2\sigma(t,t)}\right].\end{aligned}$$

In the following we consider a small deviation around the Brownian motion, by taking the mean-square displacement $\psi(t)=Kt+\epsilon\psi_1(t)+\mathcal{O}(\epsilon^2)$. We may thus assume that μ_1 is close to $x_1=0$ and μ_2 is close to $x_2=L$, leading to the ansatz:

$$\mu_i(t)=x_i+\epsilon g_i(t)+\mathcal{O}(\epsilon^2), \tag{S43}$$

$$\gamma_i(t,t')=\sigma^{(0)}(t,t')+\epsilon\gamma_i^{(1)}(t,t')+\mathcal{O}(\epsilon^2),\qquad \sigma(t,t')=\sigma^{(0)}(t,t')+\epsilon\sigma^{(1)}(t,t')+\mathcal{O}(\epsilon^2), \tag{S44}$$

$$\pi_2=(x_0+\epsilon p)/L+\mathcal{O}(\epsilon^2),\qquad \pi_1=1-\pi_2=(L-x_0-\epsilon p)/L+\mathcal{O}(\epsilon^2). \tag{S45}$$

Moreover, $\sigma^{(0)}(t,t')$ is the covariance of the Brownian motion,

$$\sigma^{(0)}(t,t') = \kappa \min(t,t'). \tag{S46}$$

At order ε^0, we find that $\mathcal{F}_i$ vanishes (as expected). At order one, introducing

$$C_i = e^{\mathrm{i}\int_0^\infty d\tau k(\tau)x_i - \frac{1}{2}\int_0^\infty \mathrm{d}\tau \int_0^\infty \mathrm{d}\tau' k(\tau)k(\tau')\sigma^{(0)}(\tau,\tau')}, \tag{S47}$$

the functional $\mathcal{F}_i([k])$ can be recast as $\mathcal{F}_i([k]) = \epsilon\, i\mathcal{F}_i^{(1)}([k]) + \mathcal{O}(\epsilon^2)$, where

$$\mathcal{F}_i^{(1)}([k]) = \int_0^\infty \mathrm{d}\tau k(\tau)\left[\left(1-\frac{x_0}{L}\right)Q_{i1}(\tau) + \frac{x_0}{L}Q_{i2}(\tau)\right] + \int_0^\infty \mathrm{d}\tau \int_0^\infty \mathrm{d}\tau' k(\tau)k(\tau')\left[\left(1-\frac{x_0}{L}\right)R_{i1}(\tau,\tau') + \frac{x_0}{L}R_{i2}(\tau,\tau')\right],$$

with

$$Q_{ij}(\tau) = \int_0^\infty \frac{\mathrm{i}\,\mathrm{d}t}{\sqrt{2\pi\kappa t}}\left\{\left[g_j(t+\tau) - g_j(t) - (x_j - x_i)\frac{\gamma_j^{(1)}(t+\tau,t) - \gamma_j^{(1)}(t,t)}{\sigma^{(0)}(t,t)}\right]e^{-\frac{(x_i-x_j)^2}{2\kappa t}} - (x_0 - x_i)\Delta(t,\tau)e^{-\frac{(x_0-x_i)^2}{2\kappa t}}\right\}, \tag{S48}$$

where

$$\Delta(t,\tau) = [\sigma^{(1)}(t,t) - \sigma^{(1)}(t+\tau,t)]/\sigma^{(0)}(t,t), \tag{S49}$$

and the value of R_{ij} is:

$$\begin{aligned} R_{ij}(\tau,\tau') = -\int_0^\infty \frac{\mathrm{d}t}{\sqrt{8\pi\kappa t}}\Big\{&\left[\gamma_j^{(1)}(t+\tau,t+\tau') - \gamma_j^{(1)}(t+\tau,t) - \gamma_j^{(1)}(t,t+\tau') + \gamma_j^{(1)}(t,t) - \sigma^{(1)}(\tau,\tau')\right]e^{-\frac{(x_i-x_j)^2}{2\kappa t}} \\ &- \left[\sigma^{(1)}(t+\tau,t+\tau') - \sigma^{(1)}(t+\tau,t) - \sigma^{(1)}(t,t+\tau') + \sigma^{(1)}(t,t) - \sigma^{(1)}(\tau,\tau')\right]e^{-\frac{(x_i-x_0)^2}{2\kappa t}}\Big\}. \end{aligned} \tag{S50}$$

Now, we show that we can find the functions $\gamma_j^{(1)}$ and g_j so that $\mathcal{F}_i([k])$ vanishes for all $[k(\tau)]$ at order ε, meaning that our theory will be exact at order ε. First, we note that, since $x(t)$ has stationary increments, σ satisfies the relation (S2) and therefore

$$\sigma(t+\tau,t+\tau') - \sigma(t+\tau,t) - \sigma(t,t+\tau') + \sigma(t,t) = \sigma(\tau,\tau'), \tag{S51}$$

and this is true at all orders of ε. Hence, if one choses $\gamma_j^{(1)}(t,t') = \sigma^{(1)}(t,t')$, then one sees that all $R_{ij} = 0$ in Eq. (S50). As a consequence, all terms that are quadratic in $k(\tau)$ in the definition of $\mathcal{F}_i^{(1)}$ vanish with this choice of $\gamma_j^{(1)}(t,t')$, meaning that the stationary covariance approximation is exact at first order. Now, for convenience let us write $g_1 = -f$ and $g_2 = g$. The terms of $\mathcal{F}_i^{(1)}$ that are linear in k vanish if f and g satisfy the integral equations

$$\int_0^\infty \frac{\mathrm{d}t}{\sqrt{t}}\left\{\frac{L-x_0}{L}[-f(t+\tau)+f(t)] + \frac{x_0}{L}e^{-\frac{L^2}{2Kt}}[g(t+\tau) - g(t) + L\Delta(t,\tau)] - x_0 e^{-\frac{x_0^2}{2Kt}}\Delta(t,\tau)\right\} = 0, \tag{S52}$$

$$\int_0^\infty \frac{\mathrm{d}t}{\sqrt{t}}\left\{\frac{L-x_0}{L}e^{-\frac{L^2}{2Kt}}[-f(t+\tau)+f(t) - L\Delta(t,\tau)] + \frac{x_0}{L}[g(t+\tau) - g(t)] - (x_0 - L)e^{-\frac{(L-x_0)^2}{2Kt}}\Delta(t,\tau)\right\} = 0. \tag{S53}$$

In the following we will obtain the functions $f(t)$ and $g(t)$ that satisfy Eqs. (S52)-(S53). Thus, $\mathcal{F}_i^{(1)}$ vanishes for all $[k]$, and we conclude that our hypothesis of Gaussianity of trajectories after the first passage, with the stationary covariance approximation, is exact at least at order $\mathcal{O}(\epsilon^1)$.

B. Explicit solution of the theory at first order

Taking derivative from Eqs. (S52)-(S53) with respect to τ gives

$$\begin{aligned} \int_0^\infty \mathrm{d}t\left[\frac{L-x_0}{L}K_1(t)f'(t+\tau) - \frac{x_0}{L}K_2(t)g'(t+\tau)\right] &= x_0 I_1(\tau), \\ \int_0^\infty \mathrm{d}t\left[\frac{L-x_0}{L}K_2(t)f'(t+\tau) - \frac{x_0}{L}K_1(t)g'(t+\tau)\right] &= (x_0 - L)I_2(\tau), \end{aligned} \tag{S54}$$

with

$$K_1(t)=\frac{1}{\sqrt{t}},\qquad K_2(t)=\frac{e^{-\frac{L^2}{2\kappa t}}}{\sqrt{t}},\qquad I_i(t)=\int_0^\infty \frac{\mathrm{d}t}{\sqrt{t}}\left[e^{-\frac{L^2}{2\kappa t}}-e^{-\frac{(x_i-x_0)^2}{2\kappa t}}\right]\partial_\tau\Delta(t,\tau). \tag{S55}$$

In order to solve the system (S54), we consider the auxiliary problem:

$$\begin{aligned}\int_0^\infty \mathrm{d}t\left[\frac{L-x_0}{L}K_1(t)f_1'(t+\tau)-\frac{x_0}{L}K_2(t)g_1'(t+\tau)\right]&=x_0A_1e^{\mathrm{i}\omega\tau},\\ \int_0^\infty \mathrm{d}t\left[\frac{L-x_0}{L}K_2(t)f_1'(t+\tau)-\frac{x_0}{L}K_1(t)g_1'(t+\tau)\right]&=(x_0-L)A_2e^{\mathrm{i}\omega\tau},\end{aligned} \tag{S56}$$

which admits the following obvious solution:

$$f_1'(t)=\frac{e^{\mathrm{i}\omega t}\,L}{L-x_0}\frac{x_0A_1\tilde{K}_1(-\mathrm{i}\omega)+(L-x_0)A_2\tilde{K}_2(-\mathrm{i}\omega)}{\tilde{K}_1^2(-\mathrm{i}\omega)-\tilde{K}_2^2(-\mathrm{i}\omega)},\quad g_1'(t)=e^{\mathrm{i}\omega t}\frac{L}{x_0}\frac{x_0A_1\tilde{K}_2(-\mathrm{i}\omega)+(L-x_0)A_2\tilde{K}_1(-\mathrm{i}\omega)}{\tilde{K}_1^2(-\mathrm{i}\omega)-\tilde{K}_2^2(-\mathrm{i}\omega)}, \tag{S57}$$

where $\tilde{h}(s)=\int_0^\infty dt h(s)e^{-st}$ represents the Laplace transform of a function h, and thus $\tilde{K}_i(-\mathrm{i}\omega)=\int_0^\infty \mathrm{d}te^{\mathrm{i}\omega t}K_i(t)$. The solution of (S54) is found by superposition; writing the functions I_i as a superposition of exponentials as $I_j(\tau)=\int_{-\infty}^\infty \frac{\mathrm{d}\omega}{2\pi}\hat{I}_j(\omega)e^{\mathrm{i}\omega\tau}$, we obtain

$$\begin{aligned}f'(t)&=\frac{L}{L-x_0}\int_{-\infty}^\infty\frac{\mathrm{d}\omega}{2\pi}\frac{x_0\hat{I}_1(\omega)\tilde{K}_1(-\mathrm{i}\omega)+(L-x_0)\hat{I}_2(\omega)\tilde{K}_2(-\mathrm{i}\omega)}{\tilde{K}_1^2(-\mathrm{i}\omega)-\tilde{K}_2^2(-\mathrm{i}\omega)}e^{\mathrm{i}\omega t},\\ g'(t)&=\frac{L}{x_0}\int_{-\infty}^\infty\frac{\mathrm{d}\omega}{2\pi}\frac{x_0\hat{I}_1(\omega)\tilde{K}_2(-\mathrm{i}\omega)+(L-x_0)\hat{I}_2(\omega)\tilde{K}_1(-\mathrm{i}\omega)}{\tilde{K}_1^2(-\mathrm{i}\omega)-\tilde{K}_2^2(-\mathrm{i}\omega)}e^{\mathrm{i}\omega t}.\end{aligned} \tag{S58}$$

Integrating these equation over t and using $f(0)=g(0)=0$ leads to

$$f(t)=\frac{x_0L}{L-x_0}\Phi_{11}(t)+L\Phi_{22}(t),\qquad g(t)=L\Phi_{21}(t)+\frac{(L-x_0)L}{x_0}\Phi_{12}(t), \tag{S59}$$

where

$$\Phi_{ij}(t)=\int_{-\infty}^\infty\frac{\mathrm{d}\omega}{2\pi}\frac{e^{\mathrm{i}\omega t}-1}{\mathrm{i}\omega}\frac{\tilde{K}_i(-\mathrm{i}\omega)\hat{I}_j(\omega)}{\tilde{K}_1^2(-\mathrm{i}\omega)-\tilde{K}_2^2(-\mathrm{i}\omega)}=\int_{-\infty}^\infty\frac{\mathrm{d}\omega}{2\pi}\frac{e^{\mathrm{i}\omega t}-1}{\mathrm{i}\omega}\frac{\tilde{K}_i(-\mathrm{i}\omega)}{\tilde{K}_1^2(-\mathrm{i}\omega)-\tilde{K}_2^2(-\mathrm{i}\omega)}\int_0^\infty \mathrm{d}x\;e^{-\mathrm{i}\omega x}I_j(x), \tag{S60}$$

where the last equality follows from the definition of $\hat{I}_j(\omega)$ as the Fourier transform of $I_j(t)$ (which we consider as vanishing for negative t). Now, let us define two functions $W_1(t)$ and $W_2(t)$ so that their Laplace transforms read:

$$\tilde{W}_i(s)=\frac{\tilde{K}_i(s)}{s[\tilde{K}_1^2(s)-\tilde{K}_2^2(s)]}. \tag{S61}$$

Changing the variables $\omega\to-\omega$ in Eq. (S60) leads to

$$\Phi_{ij}(t)=-\int_0^\infty \mathrm{d}x\int_{-\infty}^\infty\frac{\mathrm{d}\omega}{2\pi}\frac{e^{-\mathrm{i}\omega t}-1}{\mathrm{i}\omega}\tilde{W}_i(\mathrm{i}\omega)e^{\mathrm{i}\omega x}I_j(x)=\int_0^\infty \mathrm{d}x[-W_i(x-t)\theta(x-t)+W_i(x)\theta(x)]I_j(x), \tag{S62}$$

where in the second equality we have recognized the inverse Laplace transform. Finally, making shift of the variable x in the first term of the above expression and using the definition of I_j, we arrive to

$$\Phi_{ij}(t)=\frac{1}{2\kappa}\int_0^\infty \mathrm{d}x\;W_i(x)\int_0^\infty\frac{\mathrm{d}y}{y^{3/2}}\left[e^{-\frac{L^2}{2\kappa y}}-e^{-\frac{(x_j-x_0)^2}{2\kappa y}}\right][\psi_1'(x+y+t)-\psi_1'(x+y)-\psi_1'(x+t)+\psi_1'(x)], \tag{S63}$$

This expression, combined with Eq. (S59), is an explicit solution for the average trajectories f and g if one specifies the value of W_i. The functions W_i can be calculated as follows. First, using (S55) and (S61) we obtain

$$\tilde{W}_1(s)=\frac{1}{\sqrt{\pi s}\left(1-e^{-\sqrt{8sL^2/\kappa}}\right)}=\frac{1}{\sqrt{\pi s}}\sum_{n=0}^\infty e^{-n\sqrt{8sL^2/\kappa}}, \tag{S64}$$

$$\tilde{W}_2(s)=\frac{e^{-\sqrt{2sL^2/\kappa}}}{\sqrt{\pi s}\left(1-e^{-\sqrt{8sL^2/\kappa}}\right)}=\frac{1}{\sqrt{\pi s}}\sum_{n=0}^\infty e^{-(n+1/2)\sqrt{8sL^2/\kappa}}, \tag{S65}$$

where we have used geometric series in order to identify the inverse Laplace transforms of $\tilde{W}_i(s)$, leading to:

$$W_1(t) = \sum_{n=0}^{\infty} \frac{e^{-2L^2n^2/(Kt)}}{\pi\sqrt{t}} = \frac{\vartheta_3\left(0, e^{-\frac{2L^2}{Kt}}\right)+1}{2\pi\sqrt{t}}, \qquad W_2(t) = \sum_{n=0}^{\infty} \frac{e^{-2L^2(n+1/2)^2/(Kt)}}{\pi\sqrt{t}} = \frac{\vartheta_2\left(0, e^{-\frac{2L^2}{Kt}}\right)}{2\pi\sqrt{t}}. \tag{S66}$$

Here, $\vartheta_k(\cdot,\cdot)$ is the Jacobi theta function of the kth kind. Finally, let us determine now the splitting probability $\pi_2 = x_0/L + \epsilon\pi_2^{(1)} + \mathcal{O}(\epsilon^2)$, at order ϵ^1 we obtain from Eq. (1) in the main text

$$\pi_2^{(1)} L = \lim_{t\to\infty}[\pi_1^{(0)} f(t) - \pi_2^{(0)} g(t)].$$

Using the above results, we obtain

$$\pi_2^{(1)} = \int_0^{\infty}\!\!\int_0^{\infty} \frac{\mathrm{d}x\mathrm{d}y}{\sqrt{y^3x}} \frac{1+\vartheta_4\left(0, e^{-\frac{L^2}{2\kappa x}}\right)}{4\pi\kappa} \left\{\frac{x_0}{L}\left[e^{-\frac{L^2}{2\kappa y}} - e^{-\frac{x_0^2}{2\kappa y}}\right] - \left(1-\frac{x_0}{L}\right)\left[e^{-\frac{L^2}{2\kappa y}} - e^{-\frac{(L-x_0)^2}{2\kappa y}}\right]\right\}[\psi_1'(x+y) - \psi_1'(x)], \tag{S67}$$

where we have used the relation $\vartheta_3(0,q) - \vartheta_2(0,q) = \vartheta_4(0, q^{1/4})$.

C. Examples

Fractional Brownian motion. This process is characterized by $\psi(t) = \kappa t^{2H} = \kappa t + \epsilon\psi_1(t) + \mathcal{O}(\epsilon^2)$, so that for $H = 1/2 + \epsilon$ we have $\psi_1(t) = 2\kappa t \ln t$. We use the notation $u = x_0/L$. The splitting probability has the structure

$$\pi_2^{(1)} = Q(u) - Q(1-u), \text{ with } Q(u) \equiv \frac{u}{\pi}\int_0^{\infty} \frac{\mathrm{d}x}{\sqrt{x}} \sum_{k=0}^{\infty} (-1)^k e^{-\frac{k^2}{x}} \int_0^{\infty} \frac{\mathrm{d}y}{y^{3/2}}\left[e^{-\frac{u^2}{y}} - e^{-\frac{1}{y}}\right] \ln\frac{x+y}{x}, \tag{S68}$$

where we have used $\vartheta_4(0, e^{-\frac{1}{x}}) = 1 + 2\sum_{k=1}^{\infty}(-1)^k e^{-\frac{k^2}{x}}$. $Q(u)$ can be calculated by integrating first over y, and then over x, and finally summing over k, leading to

$$Q(u) = 4u(1-\ln u) + (1-2u)\left[12\ln A - \frac{7}{3}\ln 2\right] - 2\ln\pi + 8\left[\psi^{(-2)}\left(\frac{u+2}{2}\right) - \psi^{(-2)}\left(\frac{u+1}{2}\right)\right], \tag{S69}$$

where $A \approx 1.28243$ is the Glaisher-Kinkelin constant and $\psi^{(-2)}(\xi) = \int_0^{\xi} \mathrm{d}z \ln\Gamma(z)$ is the generalized polygamma function. We note that the constant term can be reformulated as $\ln(A^{12}2^{-\frac{7}{3}}\pi^{-2}) = 8[\psi^{(-2)}(1/2) - \psi^{(-2)}(1)]$, hence $Q(0) = 0$. Note that the result (S69) were obtained in (55) based on other methods.

Bi-diffusive process. This process is characterized by $\psi(t) = \kappa t + A(1 - e^{-t/\tau}) = \kappa t + \epsilon\psi_1(t) + \mathcal{O}(\epsilon^2)$, so that $\psi_1(t) = \kappa\tau(1 - e^{-t/\tau})$. With this, introducing the dimensionless variables $\ell = L\sqrt{2/(K\tau)}$ and $u = x_0/L$, we obtain

$$f(t) = \frac{L[\cosh((1-u)\ell) - (1-u)\cosh(\ell) - u]}{(1-u)\ell\sinh(\ell)}(1-e^{-t/\tau}), \qquad g(t) = \frac{L[\cosh(u\ell) - u\cosh(\ell) - (1-u)]}{u\ell\sinh(\ell)}(1-e^{-t/\tau}),$$
$$\pi_2^{(1)} = \frac{[\cosh((1-u)\ell) - \cosh(u\ell) + (1-2u)(1-\cosh(\ell))]}{\ell\sinh(\ell)}.$$

VI. GENERALIZATION OF THE THEORY TO $d \geq 1$

Here we consider the case of spatial dimension $d \geq 1$. Let us first derive general formulas for the splitting probability for a random walker of position $\mathbf{r}(t) = (x_1(t), x_2(t), ..., x_d(t))$ which has non-smooth trajectories. To the difference of the $d = 1$ case, we assume that the dynamics occurs in confinement, so that the pdf of positions, which we call

$p^c(\mathbf{r},t)$, tends to a stationary value p_s^c for $t\to\infty$. The centers of the targets are located at $\mathbf{r}_1$ and $\mathbf{r}_2$, these targets have radius a and are inside a large confining volume V. We first write the generalized renewal equation:

$$p^c(\mathbf{r}_i,t)=\int_0^t dt' F(t') p^c(\mathbf{r}_i,t|\mathrm{FPT}=t'), \tag{S70}$$

where $p^c(\mathbf{r},t|\Omega)$ represents the probability density of $\mathbf{r}$ at t given the event Ω. Note that p^c is defined in confined space and tends at large time to the stationary value $p_s^c(\mathbf{r})$. Substracting p_s^c on both sides of the above equation leads to

$$p^c(\mathbf{r}_i,t)-p_s^c(\mathbf{r}_i)=\int_0^t dt' F(t')[p^c(\mathbf{r}_i,t|\mathrm{FPT}=t')-p_s^c(\mathbf{r}_i)]-\int_t^\infty d\tau p_s^c(\mathbf{r}_i)F(\tau). \tag{S71}$$

Now, the probability density p_π^c to observe the position $\mathbf{r}$ at time t after the FPT is defined as

$$p_\pi^c(\mathbf{r},\tau)=\int_0^\infty dt\ p^c(\mathbf{r},t+\tau|\mathrm{FPT}=\tau)F(\tau). \tag{S72}$$

We note that, using Eq. (S4) with $A=\infty$ and $g(t,t')=F(t')[p^c(\mathbf{r}_i,t|\mathrm{FPT}=t')-p_s^c(\mathbf{r}_i)]$, we obtain

$$\begin{aligned}\int_0^\infty dt\int_0^t dt' F(t')[p^c(\mathbf{r}_i,t|\mathrm{FPT}=t')-p_s^c(\mathbf{r}_i)] &= \int_0^\infty du\int_0^t dt' F(t')[p^c(\mathbf{r}_i,t'+u|\mathrm{FPT}=t')-p_s^c(\mathbf{r}_i)]\\ &= \int_0^\infty du\ [p_\pi^c(\mathbf{r}_i,u)-p_s^c(\mathbf{r}_i)]\end{aligned} \tag{S73}$$

Noting also that $\langle T\rangle=\int_0^\infty dt\int_t^\infty F(t')dt'$, we see that integrating (S71) over $t\in]0,\infty[$ leads to the exact relation

$$\langle T\rangle p_s^c(\mathbf{r}_i)=\int_0^\infty dt\ [p_\pi^c(\mathbf{r}_i,t)-p^c(\mathbf{r}_i,t)]. \tag{S74}$$

Partitioning over first passage to each of the targets leads to

$$p_\pi^c(\mathbf{r},t)=\pi_1 q_1^c(\mathbf{r},t)+\pi_2 q_2^c(\mathbf{r},t), \tag{S75}$$

where $q_j^c(\mathbf{r},t)$ is the probability density function (pdf) of $\mathbf{r}$ at a time t after the first passage to target j. Hence, Eq. (S74) leads to a system of equations for $\pi_1,\pi_2,\langle T\rangle$ which is completed by the relation $\pi_1+\pi_2=1$, so that

$$\pi_1=\frac{h_{22}-h_{12}}{h_{22}+h_{11}-h_{21}-h_{12}}=1-\pi_2, \tag{S76}$$

$$\frac{\langle T\rangle}{V}=\frac{h_{11}h_{22}-h_{12}h_{21}}{h_{22}+h_{11}-h_{21}-h_{12}}, \tag{S77}$$

where

$$h_{ij}=\int_0^\infty dt[q_j^c(\mathbf{r}_i,t)-p^c(\mathbf{r}_i,t)], \tag{S78}$$

We stress that the above relations are exact for non-smooth processes whose pdf p^c reaches a steady state p_s^c, as long as one uses propagators in confined space in these expressions.

To proceed further, we need to evaluate the propagators entering into the h_{ij} terms. We use the following assumptions. First, we assume the boundaries of the volume are far enough so that all propagators can be evaluated in infinite space, the results will be valid for $V\to\infty$ when all the other parameters (distances to the targets, their radii, etc) are kept constant. In this case, we can replace the propagators p^c in the expressions of h_{ij} by their values in infinite space, $p^c\simeq p$. We also assume that, in this large volume limit, the trajectories $x_d(t)$ satisfy the same properties as in $d=1$: they are Gaussian, continuous, non-smooth, with stationary increments. Assuming isotropic walk leads to $\mathrm{cov}[x_i(t),x_j(t)]=\delta_{ij}\psi(t)$ and then $\mathrm{cov}[x_i(t),x_j(t')]=\delta_{ij}\sigma(t,t')$. We again assume that $\psi(t)\sim t^{2H}$ at long times. Note that since the MSD $\psi(t)$ is defined in free space, this divergence at long times is not contradictory with the fact that the random walk occurs in confined space; in other words the MSD in confined space will saturate at times at which the boundaries can be reached. Of note, when $d=1$, Eq. (S76) provides an alternative evaluation of π_2 to Eq. (1); it turns out that using both equations (S76) or (1) lead to the same results, as controlled on Fig. S4.

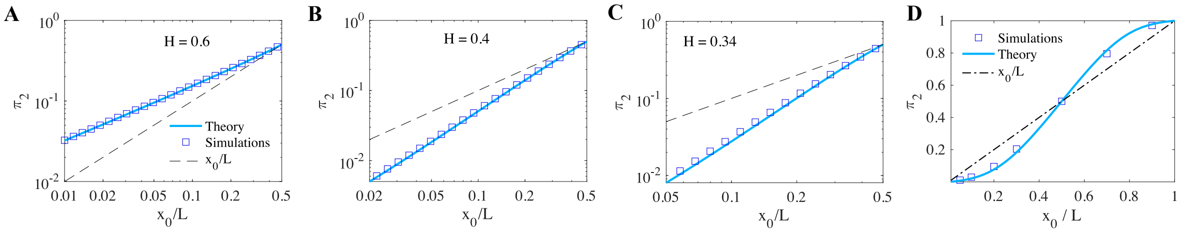

FIG. S4. **Splitting probabilities for the fractional Brownian motion and the bidiffusive process in one dimension.** The represented quantities are the same as in Fig. 2**A-D** when π_2 is evaluated with (S76) rather than with Eq. (2).

Second, we use here a decoupling approximation, by assuming that $q_i(\mathbf{r},t)$ is equal to the pdf of positions after the FPT to target i when only target i is present, in the single target problem. In this approximation, one neglects the fact that some trajectories may have touched the other target first and should not be taken into account to calculate $q_i(\mathbf{r},t)$, we expect that this approximation is valid when the distance between the targets is not too small.

Let us now focus on the $d=2$ case, the notations describing the geometry of the targets and the initial position are specified on Fig. S5. We focus temporarily on the single target problem, focusing on target i. The initial position is $\mathbf{r}_0$ and the angle between $(\mathbf{r}_0-\mathbf{r}_i)$ with respect to the x axis is $\theta_0^{(i)}$. If the position at the target surface when the target is hit is $\mathbf{r}_s$, we define θ_i the angle between $\mathbf{r}_s-\mathbf{r}_i$ and the x axis. We call $\Pi_i(\theta_i)$ the pdf of θ_i. We use the additional approximation that, after a FPT event with entrance angle θ_i (and entrance position $\mathbf{r}_s(\theta_i)$), the average trajectory after the FPT is $\mathbf{r}_i+\mu_i(t)\hat{\mathbf{u}}(\theta)$, where $\hat{\mathbf{u}}(\theta)$ is the unit vector oriented in the direction θ. Here $\mu_i(t)$ is assumed to be independent of θ_i. In these conditions, in the single target problem with only target i, one has

$$\frac{\langle T\rangle_i}{V}=\int_0^\infty dt[q_i(\mathbf{r}^*,t)-p(\mathbf{r}^*,t)]=\int_0^\infty dt\left\{\int_{-\pi}^{\pi}d\theta_i\ \Pi_i(\theta_i)\frac{e^{-\frac{[\mathbf{r}^*-\mu_i\hat{\mathbf{u}}(\theta_i)]^2}{2\psi}}}{2\pi\psi(t)}-\frac{e^{-\frac{[\mathbf{r}^*-x_0\hat{\mathbf{u}}(\theta_0^{(i)})]^2}{2\psi(t)}}}{2\pi\psi(t)}\right\}, \tag{S79}$$

for any $\mathbf{r}^*$ inside the target. Here, $\langle T\rangle_i$ is the mean FPT to target i in the single target problem. Taking $\mathbf{r}^*=a\,e_r(\theta^*)$, multiplying by $\cos(\theta^*-\theta_0^{(i)})$ and integrating over θ^* leads

$$0=\int_0^\infty dt\int_0^{2\pi}d\theta^*\cos(\theta^*-\theta_0^{(i)})\left\{\int_{-\pi}^{\pi}d\theta_i\ \Pi_i(\theta_i)\frac{e^{-\frac{a^2+\mu_i^2-2a\mu_i\cos(\theta_i-\theta^*)}{2\psi(t)}}}{2\pi\psi(t)}-\frac{e^{-\frac{a^2+x_0^2-2ax_0\cos(\theta^*-\theta_0^{(i)})}{2\psi(t)}}}{2\pi\psi(t)}\right\}. \tag{S80}$$

The integration over θ^* leads to

$$0=\int_0^\infty dt\left\{\int_{-\pi}^{\pi}d\theta_i\ \Pi_i(\theta_i)\frac{e^{-\frac{(a-\mu_i)^2}{2\psi(t)}}}{\psi(t)}\tilde{I}_1\left(\frac{a\mu_i}{}\right)\cos(\theta_i-\theta_0^{(i)})-\frac{e^{-\frac{(a-x_0)^2}{2\psi(t)}}}{\psi(t)}\tilde{I}_1\left(\frac{ax_0}{}\right)\right\}, \tag{S81}$$

where $\tilde{I}_1(x)=e^{-x}I_1(x)$, with I_1 the modified Bessel function of first kind. As a consequence, we find

$$\langle\cos(\theta_i-\theta_0^{(i)})\rangle_{\Pi_i}=\frac{\int_0^\infty dt\ \frac{e^{-\frac{(a-x_0)^2}{2\psi(t)}}}{\psi(t)}\tilde{I}_1\left(\frac{ax_0}{}\right)}{\int_0^\infty dt\ \frac{e^{-\frac{(a-\mu_i)^2}{2\psi(t)}}}{\psi(t)}\tilde{I}_1\left(\frac{a\mu_i}{}\right)}. \tag{S82}$$

Now, we will use the ansatz

$$\Pi_i(\theta_i)=\frac{e^{\alpha_i\cos(\theta_i-\theta_0^{(i)})}}{2\pi I_0(\alpha_i)}, \tag{S83}$$

which is one of the most simple function that is $2\pi-$periodic and always positive, and is also suggested by simulation results, see Fig. S6 . We obtain

$$\langle\cos(\theta-\cos\theta_0^{(i)})\rangle_{\Pi_i}=\frac{I_1(\alpha_i)}{I_0(\alpha_i)}. \tag{S84}$$

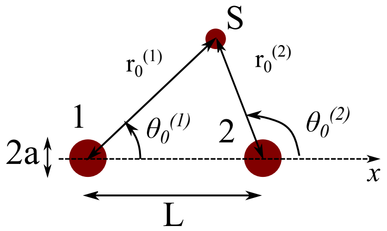

FIG. S5. **Notations for the geometry of the initial position and the target locations in the 2D problem.**

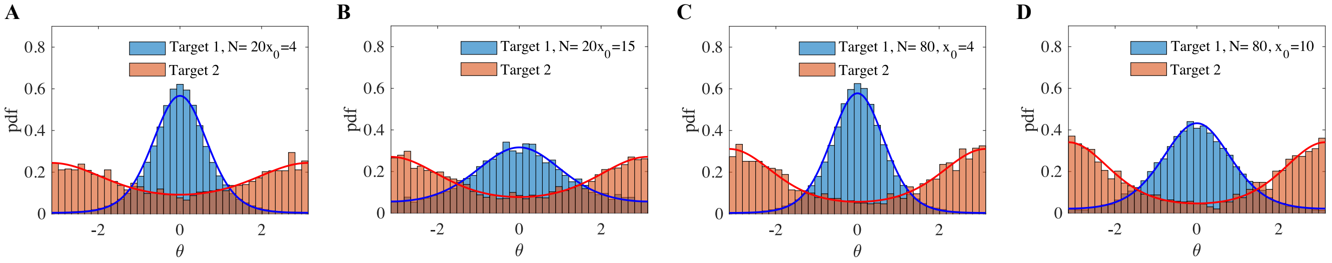

FIG. S6. **Control of the validity of the ansatz of Eq. (S83) for the system of Fig. 4(b) where the random walker is the first monomer of a Rouse polymer of N monomers.** We show the histograms of the entrance angle θ at the first passage to target 1 or 2, and a fit with the form (S83). Parameters: **A**: $N = 20$, $x_0 = 4$; **B**: $N = 20$, $x_0 = 15$; **C**: $N = 80$, $x_0 = 4$; **D**: $N = 80$, $x_0 = 10$.

As a consequence, for any position $\mathbf{r}$ so that the angle between $\mathbf{r} - \mathbf{r}_i$ and the x axis is θ, at a distance r from target i, one has

$$q_i(\mathbf{r} = \mathbf{r}_i + r\hat{\mathbf{u}}(\theta), t) = \int_0^{2\pi} d\theta_i \Pi_i(\theta_i) \frac{e^{-\frac{[r\hat{\mathbf{u}}(\theta) - \mu_i \hat{\mathbf{u}}(\theta_i)]^2}{2\psi}}}{2\pi\psi} = \int_0^{2\pi} d\theta_i \frac{e^{\alpha_i \cos(\theta_i - \theta_0^{(i)})}}{2\pi I_0(\alpha_i)} \times \frac{e^{-\frac{r^2 + \mu_i^2 - 2r\mu_i \cos(\theta - \theta_i)}{2\psi}}}{2\pi\psi}$$
$$= \int_0^{\infty} \frac{dt}{2\pi\psi} \frac{e^{-\frac{r^2+\mu_i^2}{2\psi}} I_0 \left[\sqrt{\left(\alpha_i + \frac{r\cos(\theta - \theta_0^{(i)})\mu_i}{\psi}\right)^2 + \left(\frac{r\sin(\theta - \theta_0^{(i)})\mu_i}{\psi}\right)^2} \right]}{I_0(\alpha_i)}. \quad \text{(S85)}$$

where we have used $\int_0^{2\pi} dt e^{a\cos t + b \sin t} = 2\pi I_0(\sqrt{a^2 + b^2})$. The procedure to evaluate π_2 is the following: first, we evaluate $\mu_1(t)$ and $\mu_2(t)$ for the single target problem, see (43), then we find α_1 and α_2 using (S84) and (S82), and finally we use the above expression to evaluate the h_{ij} in Eqs. (S76) and (S78).
